# Spring viraemia of carp virus modulates the time-dependent unfolded protein response to facilitate viral replication

**DOI:** 10.3389/fimmu.2025.1576758

**Published:** 2025-04-03

**Authors:** Alejandro Romero, Antonio Figueras, Beatriz Novoa

**Affiliations:** Instituto de Investigaciones Marinas Spanish National Research Council (CSIC), Vigo, Spain

**Keywords:** viral infection, SVCV, endoplasmic reticulum (ER), unfolded protein response (UPR), antiviral activity, viral replication, immune response

## Abstract

**Introduction:**

The spring viraemia of carp virus (SVCV) poses a significant threat to global aquaculture, yet effective antiviral drugs and vaccines remain unavailable. Understanding the interplay between host-pathogen interactions and SVCV replication is crucial for devising preventive strategies.

**Methods:**

ZF4 cells were exposed to UV-inactivated SVCV or live SVCV at different multiplicities of infection, and the modulation of the unfolded protein response (UPR) was assayed by qPCR at different times. Moreover, ZF4 cells were treated with several UPR modulators to investigate their effect on viral replication. The UPR was also modulated *in vivo* in zebrafish larvae, and its impact on the survival against SVCV infection was evaluated.

**Results and conclusions:**

This study reveals how SVCV exploits the host’s UPR to facilitate its replication. SVCV targets the immunoglobulin heavy chain-binding protein (BiP) and the activating transcription factor 4 (ATF4) during early infection to enhance viral RNA synthesis and translation. At later stages, activation of the BiP, the PKR-like ER kinase (PERK), and the inositol-requiring enzyme 1 alpha (IRE1α) pathways supports the release of viral progeny and induces cellular processes, including immune responses and apoptotic cell death. Furthermore, the data demonstrate that modulating UPR pathways, particularly ATF6 and PERK, significantly affect viral replication, providing a novel avenue for antiviral drug development. Preliminary *in vivo* studies suggest the feasibility of chemically modulating the UPR to combat SVCV, though optimizing administration conditions to maximize efficacy while minimizing side effects warrants further investigation. These findings offer critical insights into the molecular mechanisms underlying SVCV pathogenesis and highlight promising targets for therapeutic intervention.

## 1 Introduction

The spring viraemia of carp virus (SVCV) is an OIE-listed rhabdovirus responsible for high mortalities of cultured cyprinid fish in Europe, America and several Asian countries (1). Like other rhabdovirus, SVCV is a bullet-shaped negative-stranded enveloped RNA virus with a genome of ∼11 kb. It codifies five structural proteins organized in the order typical of rhabdoviruses: a nucleoprotein (N), phosphoprotein (P), matrix protein (M), glycoprotein (G) and RNA-dependent RNA polymerase (L) (2). Viral replication occurs in the cytoplasm, where the endoplasmic reticulum (ER) plays a vital role in its life cycle (3). Although it is not known for SVCV infection, a profound impact on ER functions has been described in several viral groups (4–7) including rhabdovirus such as the viral hemorrhagic septicemia virus (VHSV), the rabies virus, the vesicular stomatitis virus (VSV), or the Maraba virus (8–10).

The ER is a significant site for protein synthesis, folding, and transport of secretory and membrane proteins. It is also involved in the biosynthesis of phospholipids, cholesterol and steroids, the metabolism of carbohydrates, detoxification reactions and intracellular calcium storage (11). Perturbation of ER functions is induced by pathophysiological conditions, disease or exposure to environmental stressors and results in the production and aggregation of misfolded proteins and the subsequent activation of the unfolded protein response (UPR) (12). Three ER-resident transmembrane proteins monitor the quantity and quality of the proteins within the ER lumen: PKR-like ER kinase (PERK), activating transcription factor 6 (ATF6), and the inositol-requiring enzyme 1 alpha (IRE1α) (4, 13). They are usually inactivated by the attachment of the master regulator chaperone immunoglobulin heavy chain-binding protein (BiP). Under ER stress, the BiP chaperone is released from the sensors and attaches to the misfolded proteins. The three UPR pathways are now sequentially activated to maintain ER homeostasis by increasing protein-folding activity, reducing global transcription and translation, and clearing misfolded proteins (13–15). The activation of PERK induces the phosphorylation of the eukaryotic initiation factor 2 alpha (eIF2α), which reduces the load of misfolded proteins in the ER by a general blocking of the mRNA translation. However, phosphorylated eIF2α induces the translocation to the nucleus of the transcription factor 4 (ATF4), leading to the increment in the expression of, among others genes, the growth arrest and DNA damage-inducible 34 gene (GADD34), as well as BCL-2 (12, 13). GADD34 can restore the protein synthesis by de-phosphorylating the eIF2α. The ATF6 pathway is activated once this protein is transported from the ER to the Golgi apparatus and cleavage by site-1 and site-2 proteases to release the active form of ATF6 (15). ATF6 boosts the folding activity and the protein degradation by increasing the levels of chaperons such as calnexin (CANX), calreticulin (CAL), glucose-regulated protein, 94kD (GRP94) and the protein disulfide isomerase family A, member 6 (PDIa6) and ER-associated degradation components (12, 13). The activation of IRE1α allows its dimerization and autophosphorylation, which cleavages the X box-binding protein 1 (XBP1) mRNA to remove a short intron by the active C-terminal ribonuclease (RNase) domain. The generated XBP1-spliced (XBP1s) is translocated to the nucleus. It induces the expression of genes involved in protein folding, such as the DnaJ homolog, subfamily C, member 3 (DNAjC3), protein degradation, such as the ER degradation enhancer, mannosidase alpha-like 1 (EDEM1), lipid biosynthesis and cytokine production (16). IRE1α can also degrade mRNAs or microRNAs in a process called regulated IRE1-dependent decay (RIDD) that lowers the abundance of mRNA. Misfolded proteins are finally exported to the cytoplasm, degraded by the proteasome in a process called ER-associated degradation (ERAD) and cleaned by autophagy (17). When the damage to the ER is severe or the UPR is prolonged, the cells die by apoptosis (18). At this point, inflammatory reactions and immune processes are also induced by the activation of NF-kB (19–21). The UPR transducers can be selectively modulated by specific chemicals (22). The natural antibiotic tunicamycin induces the UPR by inhibiting N-linked glycosylation and affecting the maturation, displacement and accumulation of proteins in the ER. The PERK pathway can be interfered by the inhibitor GSK2606414 and the guanabenz (22–25). The ATF6 protein can be blocked in the ER membrane by ceapins (22, 26). Moreover, the kinase and the RNase activity of the IRE1α can be inhibited by APY29 and 4μ8C, respectively (27–29).

During SVCV replication, high amounts of viral proteins are produced. Viral glycoproteins suffer post-translational modifications in the ER and are transported by cellular chaperones to the Golgi apparatus and the plasma membrane (30). In this scenario, it is plausible that the nascent SVCV proteins accumulate in the lumen of ER, exceeding its folding capacity, thereby perturbing the normal cellular function of ER and activating the UPR as it has been described for the rhabdovirus VHSV (10, 31) and other enveloped viruses (6, 19, 21).

The activation of the UPR is a double-edged sword for viral replication. For example, the increment of chaperones enhances the folding of viral proteins and the induction of autophagy facilitates the release of the new viral progeny (32, 33). Moreover, ERAD proteins are used by some viruses to degrade host components with anti-viral activity (34). In contrast, the ER stress induces an innate and adaptive immune response and the inflammatory and type I interferon response can affect the cell survival and viral replication (19, 34). As a consequence, both enveloped and non-enveloped viruses have developed several specific mechanisms to modulate the anti-viral activities of the UPR. Viruses control cell stress and metabolic pathways to avoid the disruption of their viral replication by regulating the three signaling pathways of the UPR in a time-dependent manner (6, 19, 21, 34–36).

Whether and how SVCV impact the UPR in infected cells is not described. Understanding how this aquatic virus manipulates this response opens the possibility of developing new antiviral strategies by pharmaceutical targeting of the UPR as it has recently been explored in several human infections such as Chikungunya virus or Coronavirus infections (37–39). In the present study, we analyze the influence of SVCV on ER stress in the three arms of the UPR pathway and demonstrate how this virus differentially modulates the host UPR in a time-dependent manner to facilitate virus replication. We first proved that the active replication of SVCV inhibited the UPR during the early stages of infection, but it was activated at later stages. Next, we explored the modulatory activity of several chemical compounds to interfere with the UPR. Lastly, we evaluated the antiviral effect of the modulators. Our findings have implications for developing new antivirals against SVCV since intensive research has been done to develop effective curative and preventive strategies for controlling SVCV with limited success (2, 40–42).

## 2 Materials and methods

### 2.1 Cell culture

The fibroblast-like cell line ZF4, derived from 1-day-old zebrafish embryos (ATTC N° CRL-2050), was cultured in Dulbecco’s modified Eagle’s medium F12 (DMEM-F12; Gibco) supplemented with 10% fetal bovine serum (FBS; Gibco), and penicillin-streptomycin (100 IU/ml and 100 μg/ml, respectively; Gibco) at 28°C. Cells were treated with 0.1% trypsin (Gibco) and dispensed into 48 and 96-well plates (Falcon) according to the different experimental designs.

### 2.2 Viral stock

The spring viremia of carp virus (SVCV) strain 56/70 (43) was propagated in ZF4 cells at 28 °C in DMEM-F12 supplemented with 2% FBS for 48 h. After infection, the virus-containing supernatant was collected, and the cellular debris was removed by centrifugation at 12,000 xg for 5 min. Aliquots were stored at -80°C. The virus titer was determined by the inoculation of serial viral dilutions on ZF4 cells and expressed as the infective dose that induces cytopathic effect (CPE) in half of the inoculated cells (TCID50/ml) as described in (44).

UV-inactivated SVCV was also generated. Aliquots of the viral stock were transferred to a 24-well tissue culture plate (200 μl per well to a maximum depth of 2 mm) and then irradiated with a UV lamp for 1.5 h inside a class II biological safety cabinet (Euroaire, TDI). Virus inactivation was confirmed by infecting additional cell cultures and titration of supernatants. Moreover, the lack of viral replication was confirmed by qPCR, as described below.

### 2.3 Viral infections

ZF4 cells seeded at 80% confluence in multi-well plates were infected with SVCV at a multiplicity of infection (MOI) of 0.1 and 0.01 at 28 °C. Control cells were treated with culture medium or UV-inactivated virus. One hour after infection, the inoculum was removed and replaced by a fresh culture medium. According to the experimental design, supernatants and cells were sampled at different time points. The concentration of viable viral particles was calculated in the supernatants by titration as previously described. Moreover, the synthesis of the viral genome and the modulation of the UPR genes were assayed by qPCR.

### 2.4 UPR modulators

Selected chemical compounds were used to modulate the tree pathways of the UPR. All the reagents were purchased from SIGMA Aldrich. Tunicamycin (TM) was used as a universal UPR inducer. The PERK-ATF4 pathway was modulated by using GSK2606414 (termed hereafter as GSK414) and guanabenz (GBZ). The IRE1α-XBP1 pathway was inhibited using the kinase active-site inhibitor APY29 and the RNase active-site inhibitor 4μ8C. The ATF6 pathway was inhibited by using the ceapin A7. All the reagents were diluted in DMSO except GBZ that was prepared in sterile water.

### 2.5 Toxicity assay

The modulators were assayed to select a non-toxic concentration. Briefly, ZF4 cells dispensed in 96-well plates were treated for 24 h with several concentrations of the modulators prepared in culture medium (TM: 5, 2.5, 1.25 and 0.625 μg/ml; ceapin A7: 1, 3, 5, 6, 9, 10 and 15 μM; GSK414: 1, 5, 10, 15 μM; GBZ: 3.125, 6.25, 12.5, 25 and 50 μM; APY29: 0.1, 1, 2, 10 μM; 4μ8C: 3.75, 7.5, 15 and 30 μM). After this period, the modulators were removed and replaced by a new fresh medium. The toxicity was evaluated at 24 h, 48 h, 72 h and 6 days by analyzing the cellular morphology by light microscopy (Nikon Eclipse TS100) and the cell viability by adding 1 mM MTT (Sigma/Fisher) and measuring the absorbance at 560 nm in a plate reader (GlowMax, Promega) after 4 h of incubation at 28°C.

### 2.6 Antiviral activity of the modulators

ZF4 cells dispensed in 24-well plates were treated for 24 h with selected non-toxic concentrations of the modulators prepared in DMEM-F12 supplemented with 2% FBS. After removing the stimulus, the cells were infected with SVCV for 1 h at a MOI=0.1; the inoculum was removed and replaced by a new culture medium. The supernatants and the cells were sampled at different time points to measure the concentration of newly infective viral particles by titration of the supernatants and to evaluate the multiplication of the viral genome and the modulation of UPR genes by qPCR.

Additionally, other stimulatory protocols were used for TM and ceapin A7. The effect of TM was also evaluated when applied just following the viral infection (at 0 hpi) and when the viral infection was established (at 24 hpi). In the case of ceapin A7, the inhibitor can be washed off from the cells to recover the transport of the ATF6 to the Golgi apparatus. Alternatively, the antiviral activity of the ceapin A7 was evaluated when the inhibitor was maintained in the culture medium after the viral infection.

The antiviral effect of combined modulators was also evaluated. In this case, only ceapin A7 (15 μM), GBZ (12.5 μM), and APY29 (2 μM) were used. Cells were treated with combined modulators for 24 h before infection. In the case of ceapin A7, the inhibitor was maintained in the culture medium after infection.

### 2.7 *In vivo* modulation of the UPR and effect of survival to SVCV infection

The modulation of the UPR *in vivo* was assayed in zebrafish larvae. Eggs were obtained by natural spawning and reared at 28°C. Animals at 3 days post fertilization (dpf) were used to conduct the *in vivo* experiments. First, we evaluated the toxicity of the TM, GBZ and ceapin A7. Three groups of 10 larvae were treated by bath for 24 h with one of the different inducers: TM (0.5, 1 and 2 μg/ml), GBZ (25 and 50 μM), and ceapin A7 (5 and 10 μM). Control animals were treated with water containing 2.5% DMSO. The mortality was evaluated over 6 days. Additionally, 4 fish treated with different concentrations of TM were sampled after 24 h of stimulation to evaluate the induction of the UPR genes by qPCR.

Finally, the antiviral activity of the *in vivo* treatment with TM, GBZ and ceapin A7 against an SVCV infection was assayed. For those experiments, three groups of 10 larvae each were placed in culture plates with 5 ml of water. Animals were treated with a bath with different stimuli for 24 h at the concentrations previously assayed. The chemicals were removed, and the fish were infected by a bath with SVCV at a final concentration of 3x10e6 TCID50/ml in 5 ml of water. The mortalities were registered during 8 days after infection. Kaplan-Meier survival curves were constructed by using GraphPad Prism 8.0/9.0 software.

### 2.8 RNA extraction, cDNA synthesis and qPCR

Total RNA from cells and larvae was isolated using the Maxwell 16 LEV robot, according to the instructions for the simple RNA Tissue kit (Promega, Madison, WI; USA). The concentration of the RNA was measured in a NanoDrop ND1000 spectrophotometer (NanoDrop Technologies, Inc., Wilmington, DE, USA), and the first-strand cDNAs were synthesized using the NZY first-strand cDNA kit (NZY Tech) following the manufacturer recommendations. Specific primers were designed according to qPCR restrictions (**Table 1**). qPCR was performed in a StepOne Plus Real-Time PCR System (Applied Biosystems) using 1 μl of cDNA in a 25-μl reaction following the Power SYBR Green qPCR Master Mix protocol (Applied Biosystems). All reactions were performed as technical triplicates, and an analysis of melting curves was performed in each reaction. The relative expression levels of the genes were normalized using the ribosomal protein large p0 gene (RPP0) as a housekeeping gene and analyzed by the Pfaffl method (45).

**Table 1:** Sequence of qPCR primers used. The efficiency of amplification is also included.

Gene name	Primer name	Sequence (5`-3`)	Efficiency
*Immunoglobulin heavy chain-binding protein (BiP)*	*bip-F*	*AAGAGGCCGAAGAGAAGGAC*	-3,25
	*bip-R*	*AGCAGCAGAGCCTCGAAATA*	
*PKR-like ER kinase (PERK)*	*perk-F*	*TGGGCTCTGAAGAGTTCGAT*	-3,52
	*perk-R*	*TGTGAGCCTTCTCCGTCTTT*	
*Activating transcription factor 4 (ATF4)*	*atf4-F*	*TTAGCGATTGCTCCGATAGC*	-3,32
	*atf4-R*	*GCTGCGGTTTTATTCTGCTC*	
*Transcription factor C/EBP homologous protein (CHOP)*	*ddit3-F*	*AAGGAAAGTGCAGGAGCTGA*	-3,4
	*ddit3-R*	*TCACGCTCTCCACAAGAAGA*	
*Growth arrest and DNA damage-inducible protein 34 (GADD34)*	*gadd34-F*	*TTCACCATCTCCACACCTCA*	-3,15
	*gadd34-R*	*CTGCCACAGCTTCATTTTGA*	
*Activating transcription factor 6 (ATF6)*	*atf6-F*	*CTGTGGTGAAACCTCCACCT*	-3,4
	*atf6-R*	*CATGGTGACCACAGGAGATG*	
*Glucose-regulated protein, 94kD (GRP94)*	*grp94-F*	*ACGTATGGAGCAGCAAGACC*	-3,52
	*grp94-R*	*CCCACACAGTCTTCTCCACC*	
*Calnexin (CANX)*	*canx-F*	*GCGAAACCAACCACCTCAAC*	-3,39
	*canx-R*	*TGTGGTAGCCGTCAACATCC*	
*Calreticulin (CAL)*	*cal-F*	*GACTGGGATGAAGACATGGA*	-3,52
	*cal-R*	*GGTTTCCACTCACCCTTGTA*	
*Protein disulfide isomerase family A, member 6 (PDIa6)*	*pdia6-F*	*GGTGGAAAGACAGGAGGCTC*	-3,48
	*dpia6-R*	*CAGCCAGACATCATCGCTCT*	
*X box binding protein-1 (total; XBP1-t)*	*xbp1t-F*	*GAGGAGCCCACAAAGTCCTC*	-3,42
	*xbp1t-R*	*CGAAGTGCTTTTTCCTCTGG*	
*X box binding protein-1 (spliced; XBP1-s)*	*xbp1s-F*	*TGTTGCGAGACAAGACGA*	-3,55
	*xbp1s-R*	*CCTGCACCTGCTGCGGACT*	
*ER degradation enhancer, mannosidase alpha-like 1 (EDEM1)*	*edem1-F*	*ATCCAAAGAAGATCGCATGG*	-3,33
	*edem1-R*	*TCTCTCCCTGAAACGCTGAT*	
*DnaJ homolog, subfamily C, member 3 (DNAjC3)*	*dnajc3-F*	*TCCCATGGATCCTGAGAGTC*	-3,67
	*dnajc3-R*	*CTCCTGTGTGTGAGGGGTCT*	
*Ribosomal protein large p 0 (RPP0)*	*rpp0-F*	*CTGAACATCTCGCCCTTCTC*	-3,19
	*rpp0-R*	*TAGCCGATCTGCAGACACAC*	
*Nucleoprotein of* sp*ring viraemia of carp virus (N-SVCV)*	*N-svcv-F*	*TGAGGTGAGTGCTGAGGATG*	-3,52
	*N-svcv-R*	*CCATCAGCAAAGTCCGGTAT*	
*Matrix protein of* sp*ring viraemia of carp virus (M-SVCV)*	*M-scvc-F*	*ATGAGGAGACACTGGCGACT*	-3,7
	*M-svcv-R*	*CTGCAGTGAGTGGGAGTGAG*	
*Glicoprotein of* sp*ring viraemia of carp virus (G-SVCV)*	*G-svcv-F*	*CGCCCCGGATTAGACTTGAT*	-3,4
	*G-svcv-R*	*TACTGATCCGAACCCTCCGA*	

### 2.9 Statistical analysis

Statistical analysis and graphs were performed using GraphPad Prism 8.0/9.0 software. The statistical significance in the increment of viral gene expression between consecutive sampling points was evaluated by unpaired student’s t-test (*P< 0.05; ** *P*< 0.01; *** *P*< 0.001). The expression of UPR genes during viral replication and during the pharmacological modulation of the three pathways was statistically analyzed by a one-way analysis of variance (ANOVA). Significant differences between the treated and control groups at the same sampling point were calculated by a pairwise *post-hoc* Tukey test (*P<0.05 and **P<0.01).

## 3 Results

### 3.1 Time course of SVCV infection

**Figure 1:**
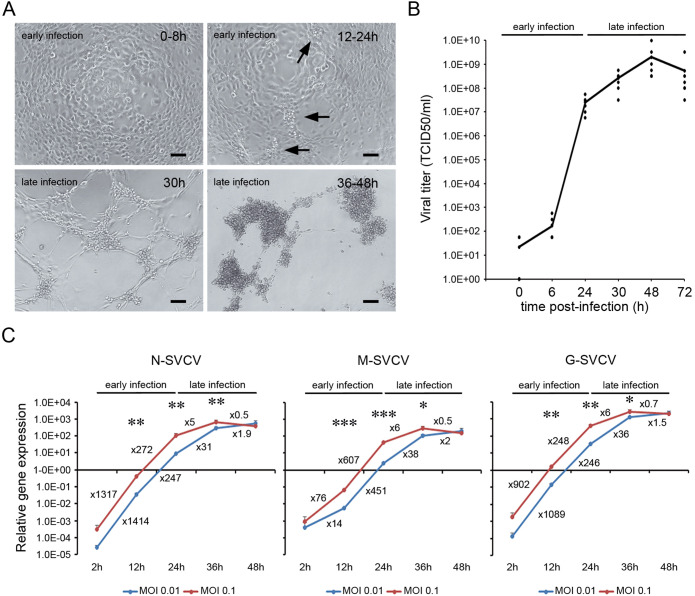
Time course of SVC viral replication. **(A)** ZF4 cells were infected with SVCV at a MOI of 0.1, and the appearance of CPE was evaluated by light microscopy. During the early phase of the infection, no CPE was observed, and only clusters of bright and round cells were observed at 24 h (arrows). The CPE appeared in the late phase of the infection, around 30 hpi and was generalized at 36-48 hpi. Scale bar = 100 μm. **(B)** Evolution of the viral titer in the supernatant of ZF4 infected cells (MOI = 0.1) at different time points (0, 6, 24, 30, 48 and 72 hpi). The dots represent the individual results obtained in six independent titrations, and the line represents the mean value. **(C)** Evolution of the synthesis of viral genes (N, M and G) at different MOIs by qPCR. Data represent the mean and SD of four independent infections. Numbers represent the relative gene expression increment compared to the previous sampling point. The statistical significance in the increment of viral gene expression between consecutive sampling points was evaluated by unpaired student’s t-test (*P< 0.05; ** P< 0.01; *** P< 0.001).

The velocity of the viral replication and the timing of the cellular events observed in the ZF4 cell depended on the infection conditions. High controlled infection conditions were applied in all the experiments to delay the appearance of CPE at least 30 h after infection (low MOI, 1h of infection and washing out the inoculum). A total CPE was obtained at 24 hpi when higher MOIs or infection times were used (data not shown). Two main steps in the viral cycle were described (**Figure 1**). An early phase of infection that extended for 24 h. During this phase, no morphological changes were observed by light microscopy. However, clusters of bright cells appeared at 24 hpi (arrows in **Figure 1A**). The late phase of the infection started at 30 hpi when the CPE was evident. The rounded cells were detached, and SVCV induced cell lysis. The virus formed well-defined plaques at 30 hpi, producing a generalized CPE between 36 and 48 hpi (**Figure 1A**). The appearance of CPE in the cells preceded the increment of the viral titer in the supernatants (**Figure 1B**). Following an initial increment of the viral concentration during the early phase, the virus titer reached its maximum value at 48 hpi (2x10e9 TCID50/ml) when the CPE was generalized and slightly decreased at 72 hpi (**Figure 1B**). The multiplication of the viral genome also followed this kinetics (**Figure 1C**). During the early phase, the N, M and G viral genes were actively transcribed, and their expression significantly increased up to 350,000 times from 2 to 24 hpi (e.g., N-SVCV). In contrast, non-significant multiplication of viral genes was detected during the late phase of the infection, with less than a 76-fold increase from 36 to 48 hpi (e.g., M-SVCV) (**Figure 1C**).

### 3.2 SVCV differentially affects each arm of the UPR during viral replication

**Figure 2:**
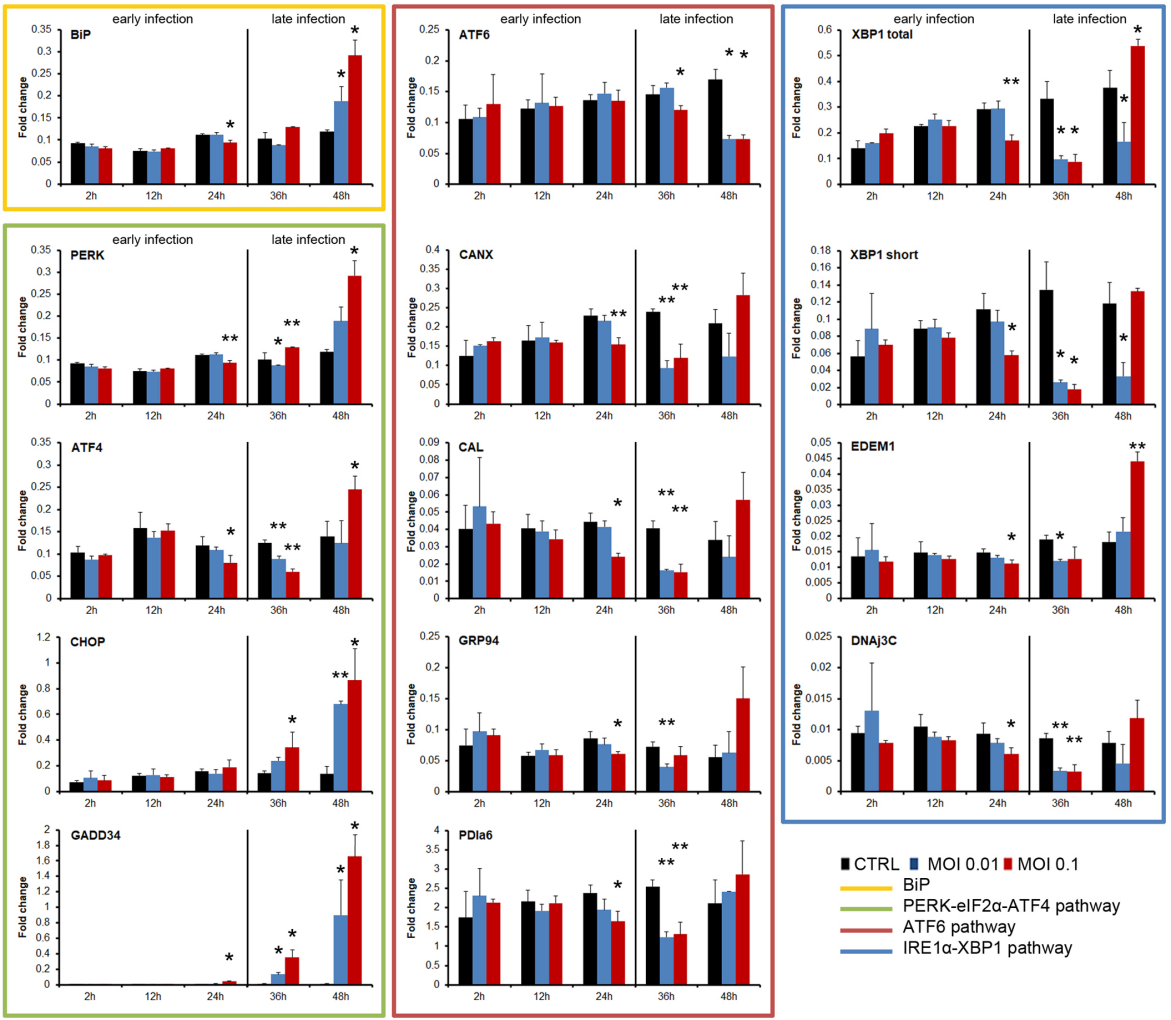
Kinetics of the UPR induction by SVCV at two different MOIs (0.1 and 0.01). The early and the late infection phases were indicated. The experiment was conducted three times using triplicates in each treatment and sampling point. A representative result of the three independent infections was presented. The gene expression in each sampling point was compared with the expression registered in the control group at the same sampling point by using one-way ANOVA with Tukey’s *post hoc* test. The significance of the difference is represented by * (*P*<0.05) and ** (*P*<0.01).

To understand the relationship of the UPR to the life cycle of SVCV, we analyzed the kinetics of the UPR during both the early phase of infection (0 to 24 hpi) and the late phase (from 30 to 48 hpi) using two different MOIs (0.1 and 0.01) (**Figure 2**). We analyzed the gene expression that initiates the pathways by qPCR, and several genes were specifically induced in each pathway.

In general, the three pathways were inhibited during the early phase of the SVCV infection and only the ATF4 and XBP1 pathways were significantly activated at the end of the infection cycle (**Figure 2**). The expression level of the BiP gene in infected cells was not different from that of the control cells during the early phase of the infection. Moreover, a significantly reduced expression was registered at 24 hpi. However, BiP expression was significantly increased at 48 hpi when total ECP was observed (**Figure 2**). SVCV modulated the PERK pathway. The PERK and ATF4 genes were significantly inhibited at 24 and 36 hpi, but they were significantly over-expressed at the end of the infection (48 hpi). The expression of the downstream genes CHOP and GADD34 was significantly increased only during all the late phase of the infection (36 and 48 hpi) regardless of the MOI used (**Figure 2**). The ATF6 gene was significantly inhibited during the late infection (36 and 48 hpi). Moreover, similar significant inhibition was registered in the genes induced after the translocation of this ATF6 factor, such as CANX, CAL, GRP94 and PDIa6 at 24 and 48 hpi (**Figure 2**). The IRE1α pathway was modified during viral replication. The expression of the XBP1 gene (total amount and short form) was significantly decreased at 24 and 30 hpi. The induced downstream genes, such as EDEM1 and DNAj3C, were also significantly inhibited simultaneously. However, at the end of the viral replication cycle, at 48 hpi, a significant increment of the XBP1 gene (total amount) and the downstream EDEM1 gene was registered (**Figure 2**).

### 3.3 UPR is activated by tunicamycin treatment

TM was used as a positive control in the analysis of the UPR modulation once a non-toxic concentration was selected (**Supplementary Figure 1**). No toxic effect was detected 24 h after the treatment. Interestingly, toxicity was registered at 48 h by MTT assay, while no alterations in cell morphology were observed. This divergence was also detected at later time points (72 h and 6 days) using 1.25 and 0.625 μg/ml of TM. However, at 72 h and 6 days, the highest doses (5 and 2.5 μg/ml) were toxic for both assays (**Supplementary Figure 1**). We selected a 2.5 μg/ml treatment to analyze UPR modulation based on those results.

**Figure 3:**
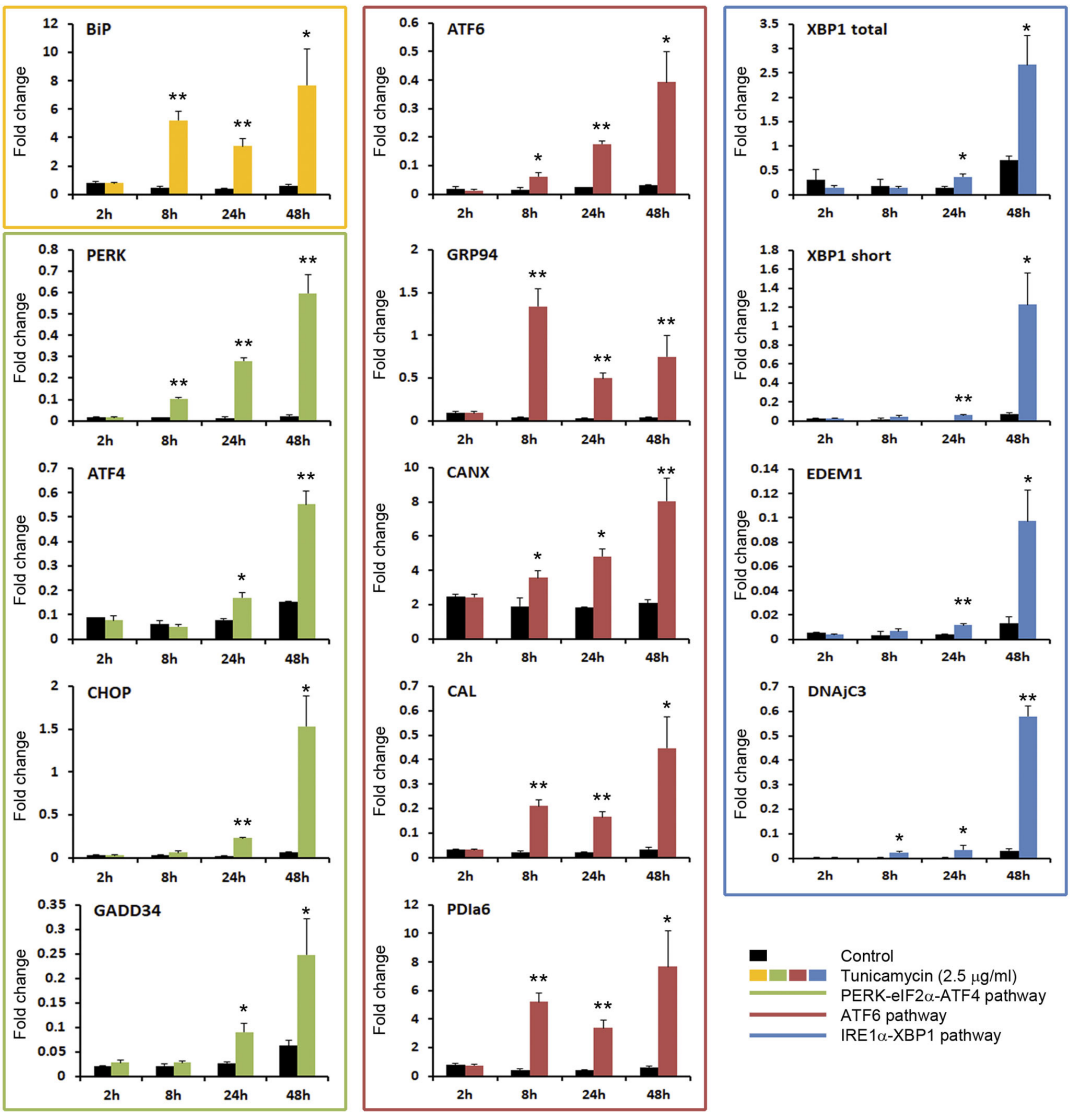
TM is a potent inducer of the UPR. The activation of the three pathways of the UPR was analyzed by qPCR. Results represent the mean and SD of 6 treated cell cultures. This experiment was conducted twice. The gene expression level obtained in TM treated cells were compared with that obtained in control cells at the same time point. Significant differences were evaluated by one-way ANOVA followed by a Tukey’s *post hoc* test and represented by * (*P*<0.05) and ** (*P*<0.01).

TM is a potent inducer of the UPR (**Figure 3**). A quick response to the TM was registered. As soon as at 8h post-stimulation, the BiP gene was highly overexpressed and maintained a significant high expression level until the end of the experiment. The ATF6 pathway was early significantly activated (8 h). The ATF6 gene and the downstream induced genes GRP94, CANX, CAL and PDIa6 maintained a significant up modulation in all sampling points (**Figure 3**). The upstream gene of the PERK pathway was also early activated, while genes induced after the translocation of the ATF4 to the nucleus, such as CHOP and GADD34, were significantly increased at 24 and 48 h (**Figure 3**). The IRE1α pathway was activated at 24 h post-stimulation when a significant increment of the XBP1 gene (total and spliced forms) and the EDEM1 and DNAjC3 genes were registered (**Figure 3**).

### 3.4 An active viral replication blocks the activation of the UPR

**Figure 4:**
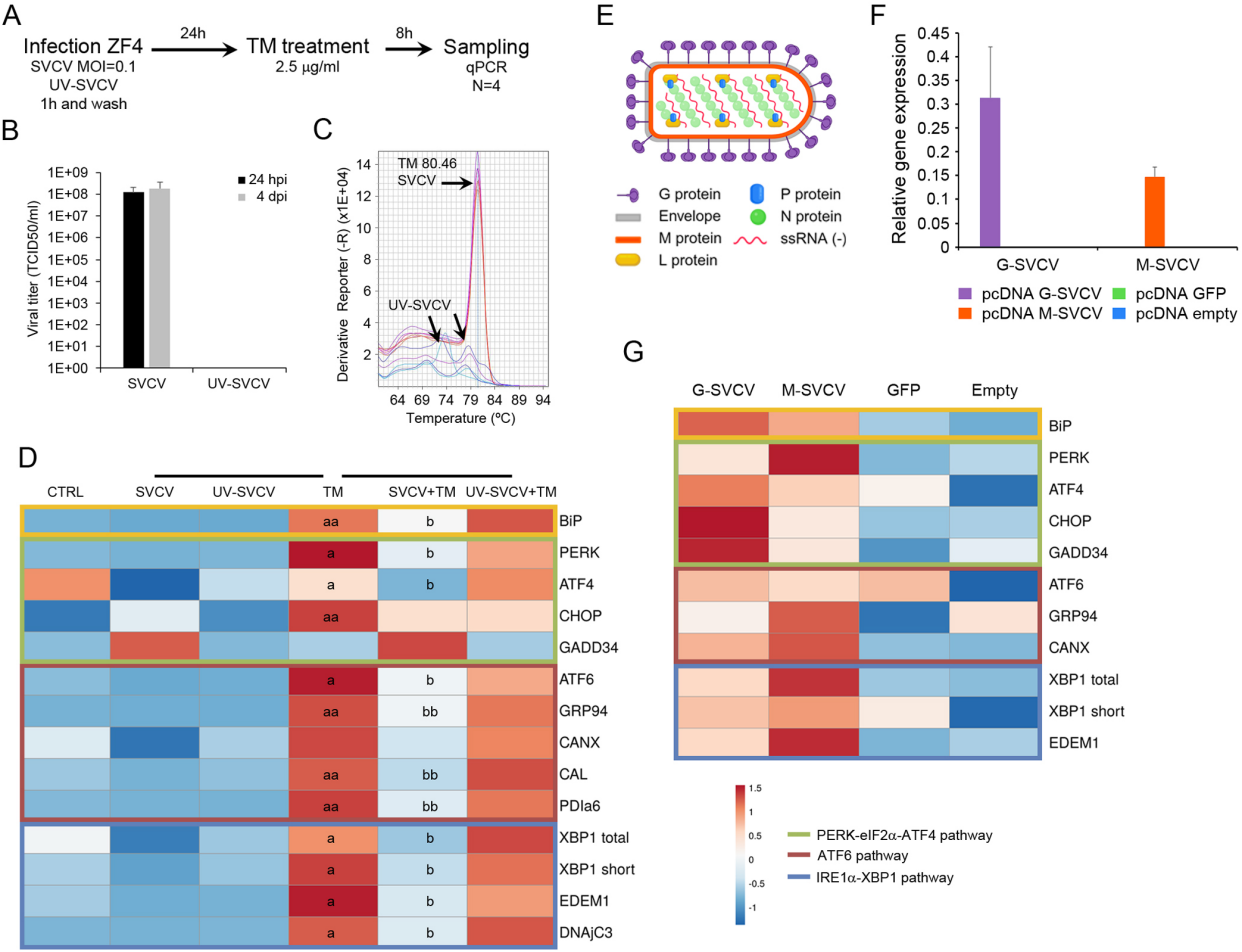
The UPR is blocked by an active viral replication. **(A)** Diagram showing the experimental procedure. **(B)** Viral titer detected in the supernatant of cells infected with SVCV and the UV-inactivated virus. Results represent the mean and SD of three independent infections. **(C)** The inactivation of the virus was also evaluated by qPCR. No viral amplification was detected. Moreover, the melting curve analysis suggests the degradation of the viral N gene after UV treatment. **(D)** A representative heat map showing how SVCV inhibits the expression of the UPR gene induced by TM. An active viral replication is needed for this inhibition. **(E)** Diagram showing the proteins conforming to the viral particles of the SVCV. **(F)** The overexpression of the viral N and G genes after transfection was confirmed by qPCR. **(G)** Expression profile of selected UPR genes in cells overexpressing the viral N and G genes. No statistically significant differences were detected between transfected and control cells. In all heatmaps, results show the mean of three individual samples. Those experiment were conducted twice. An ANOVA with Tukey’s *post hoc* test was used for the analysis. Significant differences between SVCV and TM are represented by “a” and “b”. *P*<0.05 (a and b); *P*<0.01 (aa and bb).

To further demonstrate that an active SVCV replication is needed to block the UPR, we analyzed how a preceding viral infection decrease the expression of the UPR genes induced by TM. Moreover, to exclude the possibility that the virus attachment rather than the virus infection modulated the UPR, we also used UV-inactivated SVCV. We infected ZF4 cells with SVCV (MOI=0.1), and 24 h after infection, the UPR was induced with TM. Samples were taken 8 h after treatment, and the UPR gene expression profile was analyzed by qPCR (**Figure 4A**). The absence of CPE confirmed virus inactivation after two consecutive inoculations on ZF4 cells and the lack of infective virus in the supernatant (**Figure 4B**). Moreover, no viral detection was registered by qPCR in cells treated with UV-inactivated virus. The melting curve analysis also suggested that the viral gene was degraded by the UV treatment since small random peaks with lower melting temperature than expected were obtained (**Figure 4C**).

The expression profile of the UPR genes was presented in a heatmap (**Figure 4D**). The TM treatment significantly increased almost all UPR genes compared to control cells. The infection of the cells before the TM treatment induced a significant reduction of the BiP expression, while no significant effect was detected using inactivated virus (**Figure 4D**). All the analyzed genes included in the ATF6 and the IRE1α pathways showed a similar modification of the expression profile. Only the cells previously infected with SVCV showed a significant reduction in the expression of ATF6, GRP94, CAL, PDIa6, XBP1, EDEM1 and DNAjC3 induced by the TM treatment. In the PERK pathway, the PERK, ATF4 and CHOP genes were significantly induced by TM, but only the expression of PERK and ATF4 genes was significantly inhibited by the viral replication (**Figure 4D**).

To discern if a specific viral protein is responsible for the UPR modulation rather than the whole infective virion, we overexpressed the viral nucleoprotein and the glycoprotein genes in ZF4 cells (**Figure 4E**). We analyzed the expression profile of the UPR genes. Using the plasmid pcDNA3.1-GFP, we measured 10% of transfected cells showing green fluorescence by microscopy. This percentage of transfection was enough to express both the G and the N genes at high levels (**Figure 4F**). This overexpression did not significantly modulate the expression of the UPR genes. However, a generalized higher expression of all UPR genes was detected in cells transfected with viral genes compared to control cells (**Figure 4G**). The overexpression of the GFP protein did not affect the gene expression profile since the cells showed a similar profile to that observed in cells transfected with the empty plasmid (**Figure 4G**).

### 3.5 The induction of UPR drastically affects the viral replication at early infection stages but has no effect at later ones

**Figure 5:**
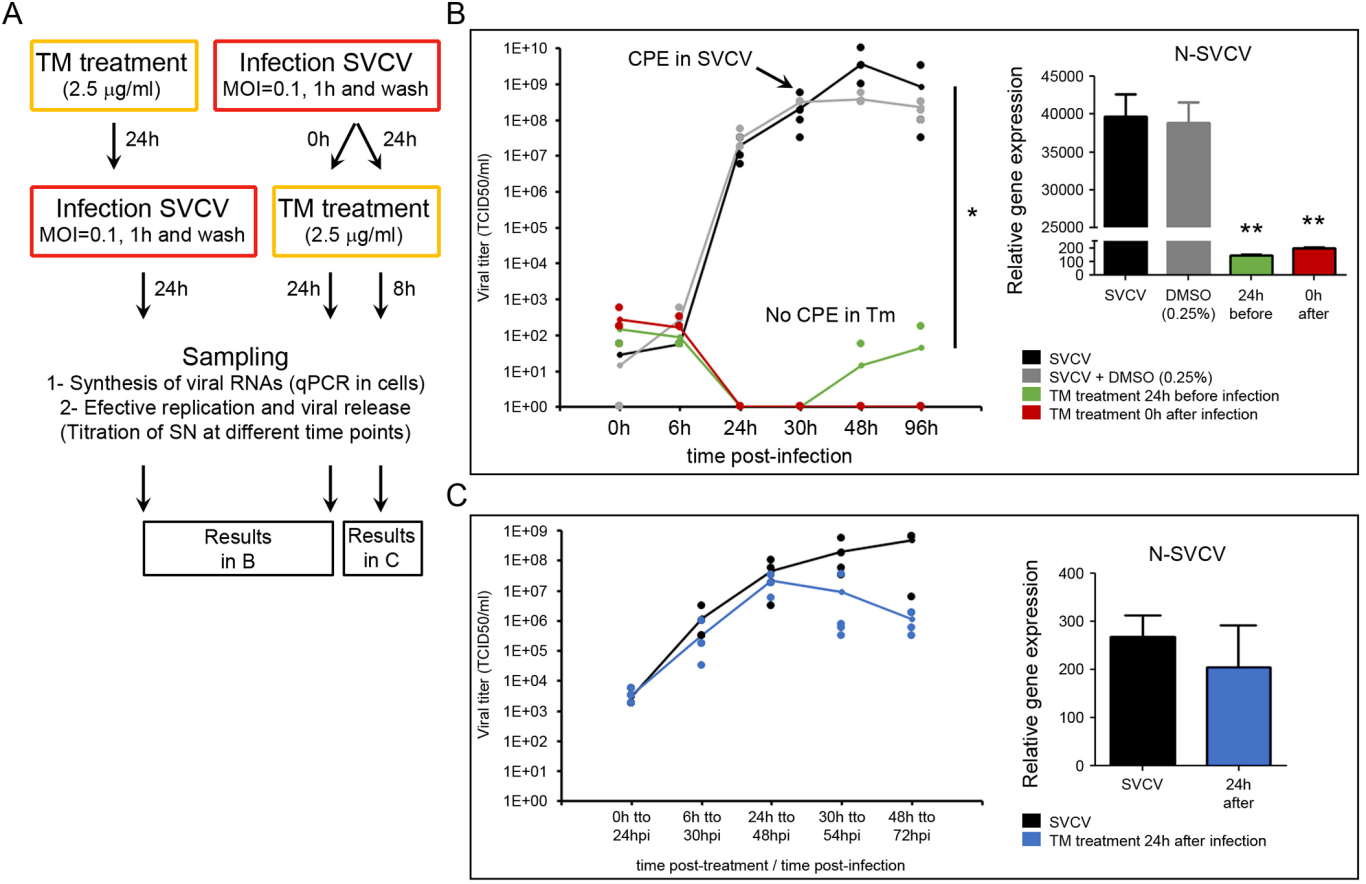
TM affects the viral replication. **(A)** Diagram showing the experimental procedures. TM was applied 24 h before and at different times after the infection (0 h and 24 h). The multiplication of the viral genome was assayed in cells by qPCR, while the production of infective viral progeny was assayed by titration of the cell supernatants. Four replicates were used in those experiments. The experiment was conducted twice. **(B)** Effect of the TM applied 24 h before the infection and immediately after the infection (0 h). **(C)** Effect of the TM applied 24 h after the infection. In the graphs, dots represent the individual results obtained in the four independent titrations, and the line represents the mean value. The amplification of the viral genome inside the cells was analyzed by qPCR in four samples. Data were analyzed by one-way ANOVA. Significant differences between TM treated and non-treated cells were analyzed by a Tukey’s *post hoc* test and represented by * (*P*<0.05) and ** (*P*<0.01).

We analyzed if a pharmacologic induction of the URP by a TM treatment can affect the viral cycle and be detrimental to the virus multiplication. TM treatment was applied before and after the infection. The effect of the TM treatment in the multiplication of the viral genome was assayed by qPCR, while the effect on the assembly of the viral proteins to obtain infective viral particles was assayed by viral titration (**Figure 5A**). A strong significant inhibition of the viral replication was obtained when cells were treated with TM 24 h before the infection and when they were treated immediately after the infection (**Figure 5B**). The titration of the supernatants revealed that infective particles were not produced during the infection. The viral titer decreased from the highest values (1x10e10 TCID50/ml) to be almost undetectable (1x10e0 TCID50/ml) when TM was applied 0 h after infection. Cells treated with TM did not show any CPE during the experiment, while the classical viral-induced CPE was evident at 30 hpi (**Figure 5B**). qPCR results also indicated that the viral genome replication was inhibited since a significant reduction in the expression of the viral N gene was registered in TM-treated cells (**Figure 5B**).

In contrast, TM did not affect the viral replication when applied once the viral infection was established after 24 hpi (**Figure 5C**). In this case, the CPE started in all the experimental groups 6 h after treatment, and the viral titer increased during the sampling points. Significant differences in the viral titer and the expression of the viral N gene were not registered between controls and TM-treated cells (**Figure 5C**).

### 3.6 The modulation of the PERK and the IRE1α pathways interfere with viral replication

The toxicity of the different modulators was evaluated to select suitable doses for functional antiviral assays. GBZ and GSK414 were used to modulate the PERK pathway and the APY29 and 4µ8C for the IRE1α pathway. None of the modulators showed toxicity at short times after treatment (24 and 48 h). At 72 h, only the highest dose of GBZ (25 µM) induced a significant reduction of the MTT assay. However, at 6 days after treatment, GBZ (in all concentrations) and APY29 and GSK414 at the highest dose (15 and 4 µM, respectively) showed toxic effects. Cells treated with 4µ8C showed no toxic effect in all the sampling times (**Supplementary Figure 1**). The concentrations selected to modulate the PERK pathway were 12.5 and 6.25 µM for GBZ and 2 and 1 µM for GSK414. The IRE1α pathway was modulated using APY29 at 2 and 1 µM and 4µ8C at 15 and 7.5 µM.

**Figure 6:**
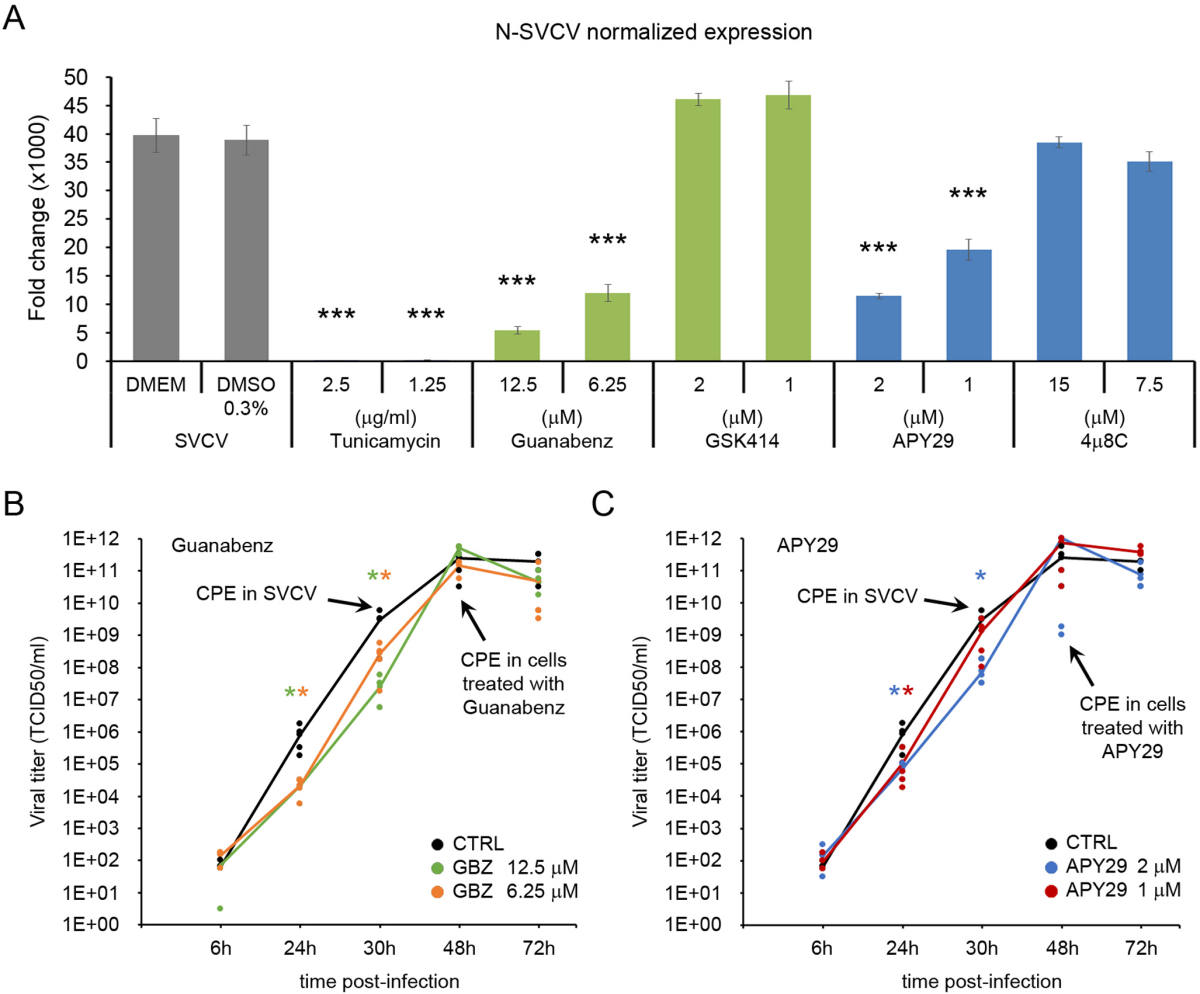
The chemical modulation of the PERK and the IRE1α pathways affects the viral replication. **(A)** Four different modulators were initially screened for their antiviral effect (GBZ, GSK414, APY29 and 4μ8C). Cells were treated with non-toxic concentrations of the modulators for 24 h and then infected with SVCV at a MOI=0.1. Viral replication was evaluated in four independent cell cultures at 24 hpi by qPCR, and the mean ± SD is presented. The release of infective viral particles was only evaluated in the cells treated with GBZ **(B)** and APY29 **(C)**. Data were analyzed by one-way ANOVA. Significant differences between infected cells treated or not with modulators were analyzed by a Tukey’s *post hoc* test and represented by * (*P*<0.05) and *** (*P*<0.001).

The multiplication of the viral genome was significantly affected when the cells were treated with GBZ and APY29 (**Figure 6A**). A significant reduction in the normalized expression of the viral N gene was obtained at 24 hpi. Treating cells with GSK414 and 4µ8C at the selected doses did not modify the viral replication at the same time point (**Figure 6A**). The generation of infective viral particles was only followed in the supernatant of cells previously treated with GBZ and APY9 (**Figures 6B** and C). Both treatments induced a delay in the viral replication. In the supernatant of cells treated with GBZ, the viral titer was significantly lower than that registered in control cells at 24 and 30 hpi in any of the selected concentrations (12.5 and 6.25 µM). Moreover, the appearance of the CPE was delayed and started to be evident at 48 hpi (**Figure 6B**). A similar response was obtained in cells treated with APY29, although the significant reduction of the viral titer at 30 hpi was only registered in cells treated with the highest concentration (2 µM). The appearance of CPE was also delayed until 48 hpi (**Figure 6C**).

The specificity of the treatment with GBZ and APY29 to modulate the PERK and the IRE1α pathways was assayed by qPCR (**Supplementary Figures 2**, **3**). The modulators were added to the culture medium, and cells were sampled after 24 h. The effect was also analyzed in cells where the UPR was induced with TM. In this case, the cells were treated with the modulators for 24 h before adding the TM (2.5 ug/ml). Cells were sampled 8 h after TM treatment. GBZ specifically modified the expression of the PERK and the ATF4 genes and induced a significant increment of both genes alone and when the UPR was activated by the TM. Interestingly, GBZ also significantly increased the XBP1 (total and spliced form) after TM activation. A significant effect was detected neither in the expression of the BiP gene nor in all genes included in the ATF6 pathway (ATF6, GRP94, CANX, PDIa6) (**Supplementary Figure 2**). The APY29 inhibited the IRE1α pathway. APY29 significantly decreased the expression of the XBP1 short and the downstream induced EDEM1 and DNAjC3 genes. However, APY29 modulates the expression of several genes included in the other activation pathways (**Supplementary Figure 3**). A significant reduction of the BiP expression level was registered when UPR was induced with TM. Inside the PERK pathway, APY29 induce a significant decrease in the PERK and ATF4 genes after TM treatment. In contrast, the GADD34 gene was significantly up modulated with and without a previous stimulation with TM. The ATF6 pathway was also significantly inhibited by the APY29 alone and in combination with TM treatment since the expression of all the analyzed genes was decreased (**Supplementary Figure 3**).

### 3.7 Blocking the ATF6 pathway delays the SVCV replication

The ATF6 pathway was inhibited by ceapin A7, which prevented the transport of the ATF6 to the Golgi apparatus during the ER stress response. First, we evaluated the toxicity of the ceapin A7 applied in a 24 h treatment and when the inhibitor was maintained in the culture medium. The cells stimulated for 24 h with ceapin A7 (9, 6, 3 and 1 μM) showed no sign of toxicity by both microscopy and MTT assay at 24 h, 48 h, 72 h and 6 days after treatment. Moreover, the inhibitor (1, 5, 10 and 15 μM) was not toxic when maintained in the culture medium at the same time points (**Supplementary Figure 1**).

The specificity of the ceapin A7 to block the ATF6 pathway was assayed by qPCR (**Supplementary Figure 4**). The inhibitor was included in the culture medium, and cells were sampled after 24 h. The inhibitory effect was also proved in cells where the UPR was induced with TM. In this case, the cells were treated with ceapin A7 for 1.5 h before adding the TM (2.5 ug/ml) in a medium containing the inhibitor. The ceapin A7 specifically inhibited the ATF6 pathway. The inhibitor did not modify the expression of the initiator ATF6 gene but affected the expression of downstream genes induced after the translocation of the ATF6 protein. A significant decreased expression of the PDIa6 and GRP94 genes was observed in cells treated with the inhibitor. However, ceapin A7 significantly reduced the expression of all the genes (PDIa6, GRP94, CANX, and CAL) when the UPR was induced by the TM treatment (**Supplementary Figure 4**). The ceapin A7 did not modify the expression of the BiP gene. The PERK pathway was not affected by the inhibitor. No significant differences in the expression of PERK, ATF4, CHOP and GADD34 genes were induced by the inhibitor when TM induced the UPR. However, the inhibitor induced a small significant increment of the CHOP gene (**Supplementary Figure 4**). The IRE1α pathway was also not affected. No changes in the expression of XBP1 (total and short forms), EDEM1 and DNAj3C genes were induced by the ceapin A7 treatment (**Supplementary Figure 4**).

**Figure 7:**
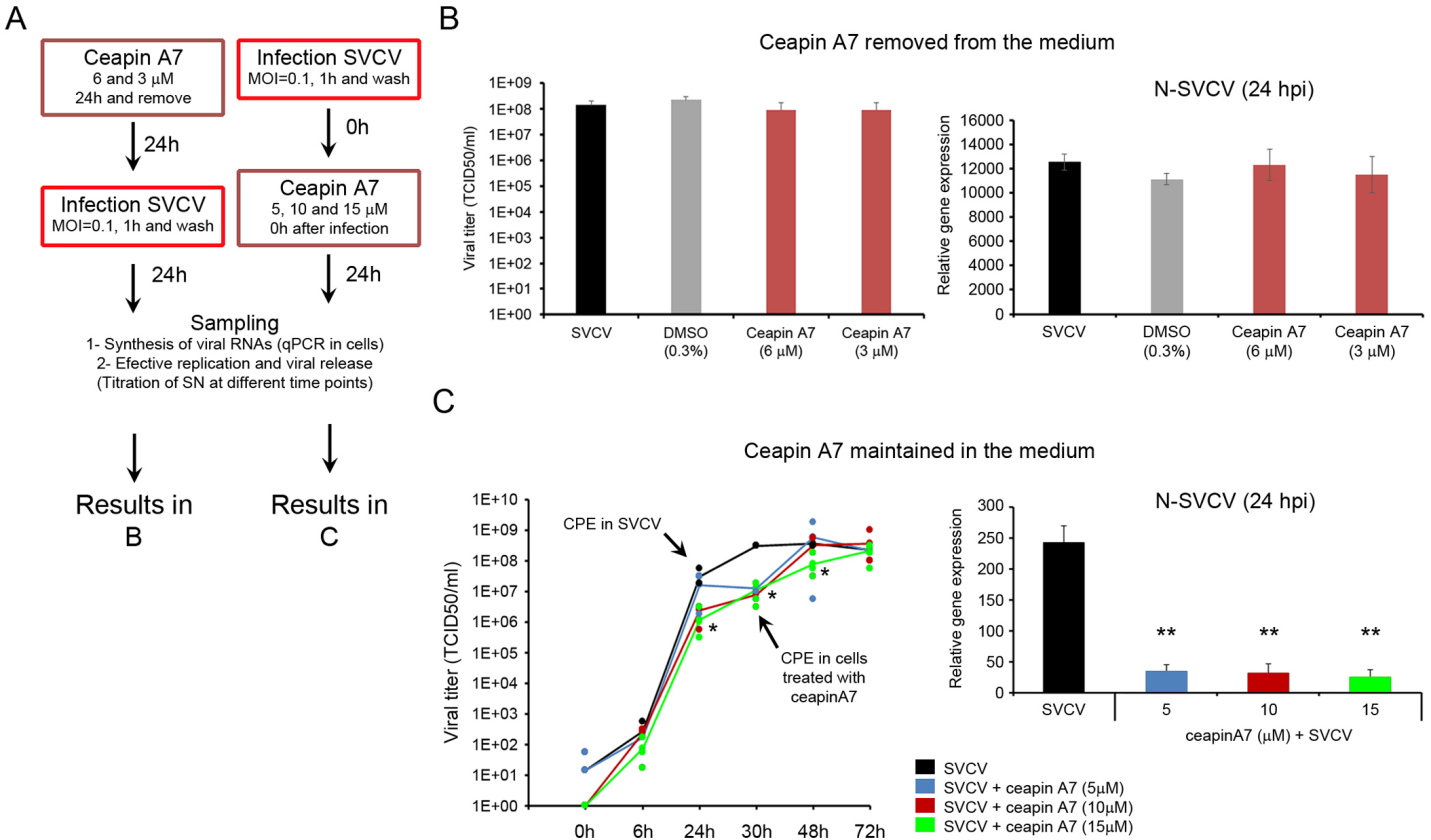
The inhibition of the ATF6 pathway delayed the viral replication. **(A)** Diagram showing the experimental procedure. The antiviral effect of the ceapin A7 was assayed after a 24 h treatment and when the inhibitor was maintained in the culture medium. **(B)** Antiviral effect of the ceapin A7 applied 24 h before the infection. No effect on the viral replication was registered by qPCR and titration. Results represent the mean and SD of four samples. **(C)** Ceapin A7 showed antiviral activity when maintained in the culture medium. A significant decrease in the expression of the viral N gene was registered by qPCR. This low number of viral copies at 24 hpi was accompanied by a delay in the appearance of the CPE until 30 hpi. Results represent the mean and SD of four independent samples. Data were analyzed by one-way ANOVA. Significant differences between infected cells treated or not with ceapin A7 were analyzed by a Tukey’s *post hoc* test and represented by * (*P*<0.05) and **(*P*<0.01).

The ceapin A7 showed antiviral activity only when the inhibitor was maintained in the culture medium. In the first approach, the inhibitor was washed out after 24 h of stimulation and the infected cells were maintained in a culture medium. (**Figure 7A**). In this model, ceapin A7 did not affect the viral replication at 24 hpi. We did not detect modifications in the viral genome replication by qPCR, and a similar concentration of infective viral particles in the supernatant was registered by titration (**Figure 7B**). However, when the inhibitor was added to the cells immediately after the virus adsorption and maintained in the cell medium, a significant modification of the viral replication was registered (**Figure 7C**). At 24 hpi the ceapin A7 (at 5, 10 and 15 μM) significantly reduced the expression of the viral N gene. Moreover, the viral titer decreased significantly when ceapin A7 was used at 10 and 15 μM at 24 and 30 hpi. This significant reduction was maintained until 48 h using the highest inhibitor dose. The appearance of CPE in the cells was delayed and began at 30 hpi. No significant differences in the viral titer were registered at later time points (48 and 72 hpi) (**Figure 7C**).

### 3.8 The combination of modulators induces a synergic antiviral effect

**Figure 8:**
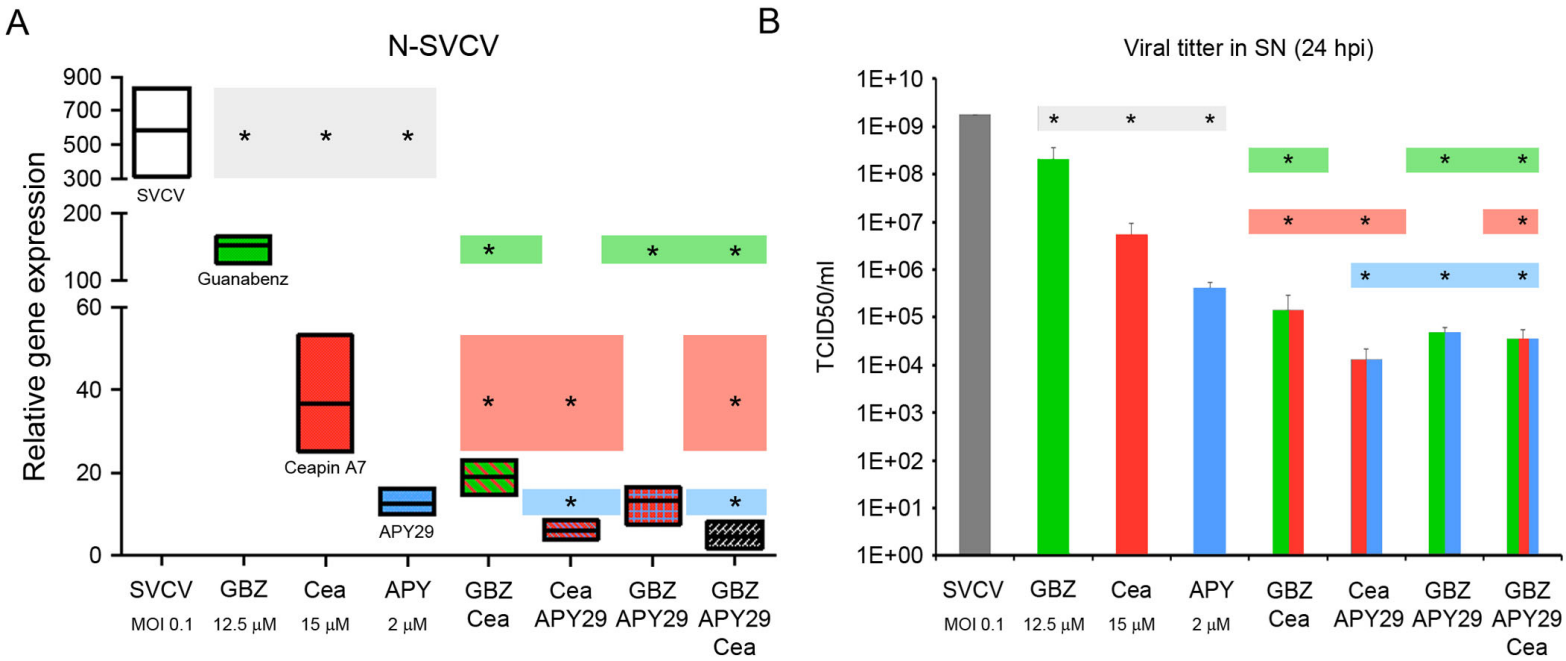
Synergic antiviral activity of the UPR modulators. **(A)** Effect on the viral genome replication analyzing the relative expression of the N gene. **(B)** Effect on the release of newly viral particles in the SN by calculating the viral titer. The results represent the mean and SD of 3 samples in all cases. Colored lines represent the N gene expression **(A)** and the viral load **(B)** and highlight the significance of the individual treatments compared to the combined ones. Two independent experiments were conducted. An ANOVA with Tukey’s *post hoc* test was used for the analysis. Significant differences between treatments are represented by * (*P*<0.05).

As we obtained previously, the individual treatments of cells with GBZ, ceapin A7 and APY29 induced a significant decrease in the transcription of the N gene and the virus released to the supernatant compared to SVCV infected cells. Interestingly, the treatment with APY29 induced the highest reduction compared to cells treated with GBZ and ceapin A7 (**Figures 8A, B**). The combination of different modulators induced a higher antiviral effect. The mixtures GBZ-ceapin A7 and APY29-ceapin A7 induced a significantly higher reduction of the N-gene expression compared with both stimuli alone. (**Figures 8A, B**). However, the three combinations significantly reduced the viral titer in the SN. The combination of the three modulators did not significantly decrease the transcription of the N gene and the viral titer compared to the double stimulations (**Figures 8A, B**).

### 3.9 The URP can be modulated *in vivo* on zebrafish larvae, but it does not affect the survival against SVCV

**Figure 9:**
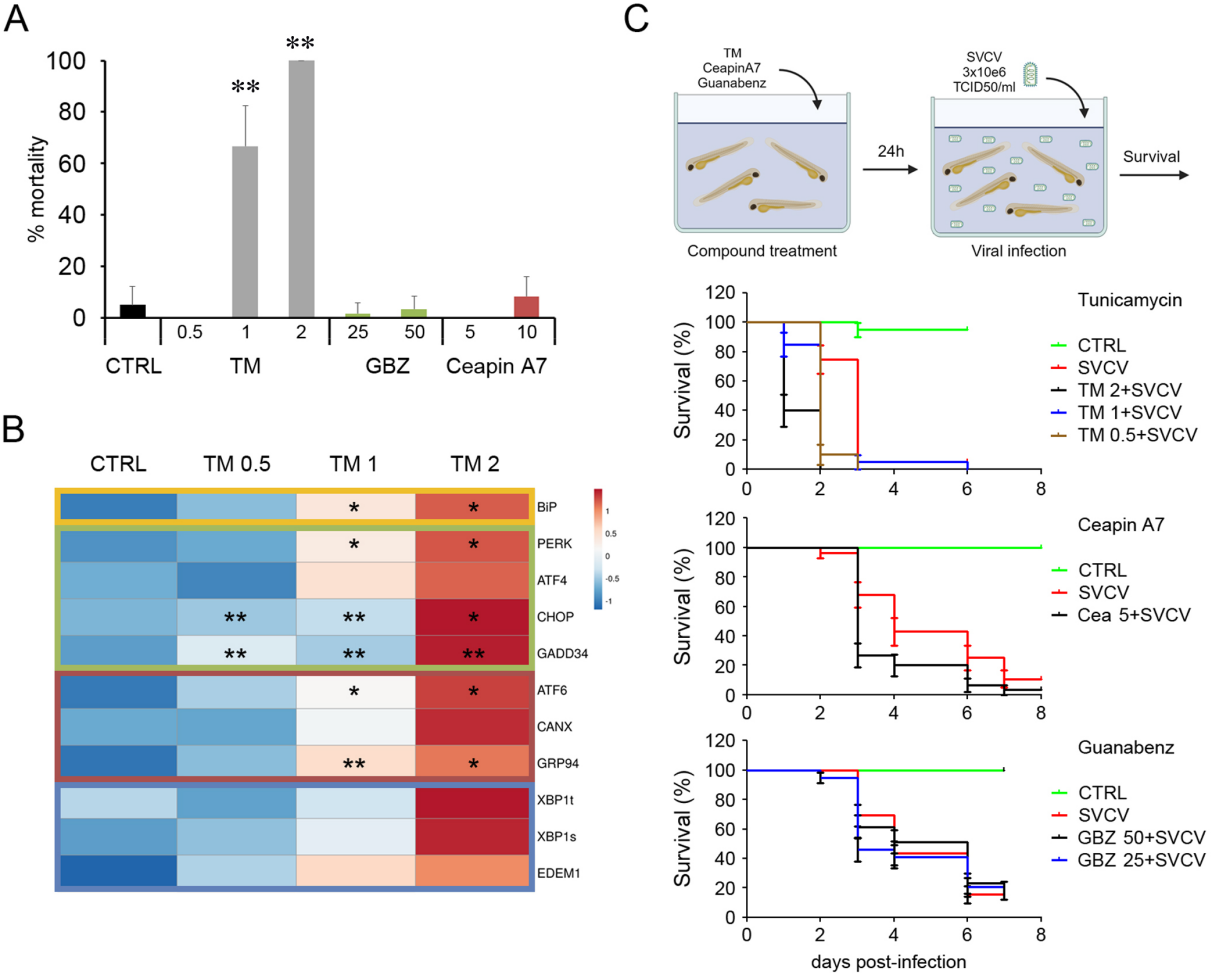
Administration *in vivo* of the UPR modulators in zebrafish larvae. **(A)** The toxicity of the modulators was assayed in 3 dpf larvae. Three groups of 10 fish were treated with different concentrations, and mortalities were registered over 6 days. Results represent the mean and SD. **(B)** The induction of the UPR was only evaluated in fish treated with TM. The heatmap represents the gene expression’s mean value registered in 4 fish. An ANOVA with Tukey’s *post hoc* test was used for the analysis. Significant differences between control and TM treated fish are represented by * (*P*<0.05) and ** (*P*<0.01). **(C)** The antiviral activity of the modulators against SVCV was also assayed *in vivo*. Thirty animals were treated with a bath with the modulators for 24 h and infected with SVCV. No significant differences in the mortality kinetics were observed in the Kaplan-Meier survival curves.

Zebrafish larvae at 3 dpf were used to conduct *in vivo* experiments. First, the toxicity of the different chemicals was assayed. The treatment with TM (1 and 2 μg/ml) induced high mortalities ranging from 60 to 100%. In contrast, GBZ and ceapin A7 at the selected doses (ceapin A7 at 5 and 10 μM and GBZ at 50 and 25 μM) were not toxic for the fish, inducing mortalities similar to that registered in the control group (**Figure 9A**). The *in vivo* modulation of the UPR was only confirmed in fish treated with TM. A general increased expression of all the UPR genes was observed when fish were treated with any dose of TM. However, differences between replicates avoid the statistical significance in several genes (**Figure 9B**). The treatment of fish larvae with the modulator did not induce changes in the survival against a lethal infection with SVCV. Moreover, the mortality kinetics registered in infected fish previously treated with any TM and ceapin A7 dose were significantly faster than those registered in non-treated fish (**Figure 9C**). None of the treatments used induced protection against SVCV when administered using the described protocol.

## 4 Discussion

**Figure 10:**
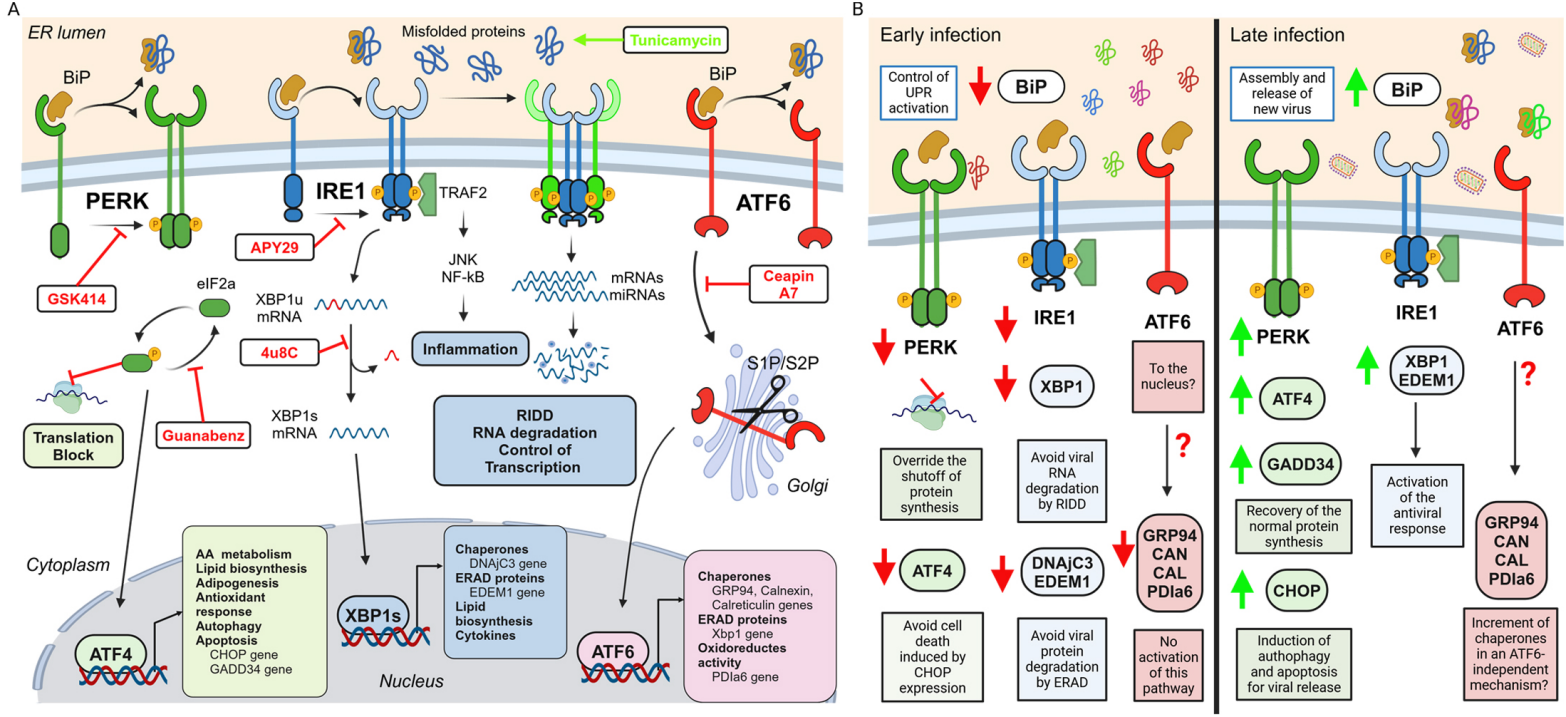
**(A)** UPR pathway. The UPR is initiated by the accumulation of misfolded proteins in the ER. They induce the dissociation of the BiP protein from the ER receptors to coordinate the activation of three different pathways. TM can induce chemical activation of this response. The PERK pathway is based on the phosphorylation of the eIF2α factor, which results in the inhibition of the ribosome assembly and the blocking of mRNA translation. Moreover, the phosphorylated eIF2α allows the translocation of the ATF4 factor to the nucleus to induce the transcription of genes related to cell survival, apoptosis, autophagy, antioxidant response lipids and amino acid metabolism. GSK414 and GBZ can chemically modulate this pathway. The activation of the IRE1α receptor causes the splicing of the XBP1 mRNA. This transcription factor targets genes encoding chaperones, genes involved in the degradation of misfolded proteins (ERAD), and the production of lipids and cytokines. Moreover, IRE1α can also induce the inflammatory response by activating the NF-kB pathway and controlling the transcription by the degradation of the mRNAs. The kinase inhibitor APY29 and the Rnase inhibitor 4μ8C modulate the IRE1 pathway. After activation, the ATF6 is fragmented by the S1P/S2P enzymes in the Golgi apparatus and translocated to the nucleus. ATF6 induces the expression of chaperone genes at the ER level (GRP94, CANX, CAL), promotes the expression of ERAD proteins, and modulates the oxidoreductase activity. The translocation of the ATF6 from the ER membrane to the Golgi apparatus can be inhibited by the ceapin A7 avoiding activating this pathway. **(B)** Modulation of the UPR pathway by SVCV infection. During the early infection, the expression of the BiP gene is inhibited to retard the activation of the UPR until the viral genome was replicated. The PERK pathway is also down modulated to override the host cell-mediated shutoff of protein synthesis and ensure a complete genome multiplication and viral protein assembly. Surprisingly, SVCV does not modulate the ATF6 pathway under our infection conditions. The inhibition of the IRE1α pathway also avoid the degradation of viral RNAs and proteins by RIDD and ERAD, respectively. At the late phase, the BiP gene is highly expressed, suggesting a critical role during the release of the new virions. At this time the GADD34 and CHOP genes belonging to the PERK pathway are highly expressed to contribute to the recovery of the normal protein synthesis and activation of apoptosis and autophagy, which are completely necessary during the release of nascent SVCV. The IRE1α pathway is also activated to induce the antiviral response. Figures were created in BioRender (BioRender.com/g23n520).

As with other viruses, the dynamic of SVCV infection depends on several factors, including virus isolate, MOI, temperature, host, age and condition, fish density and stress factors (46). A strict control of the infection process is needed to correlate the timing of the genome multiplication, the assembly of new virions and their release with the specific modulation of the three arms of the UPR. Using a low MOI and a short infection time, we extended the viral replication up to 48-72 h, obtaining an adequate time frame for the analysis of the UPR. Under our experimental conditions, the SVCV life cycle was divided into an early and a late phase of infection. The switch between the active genome transcription (early phase) and the viral replication (late phase) seems to be regulated by the levels of the N protein as it was described for other rhabdoviruses such as rabies virus (3, 47). By kinetic studies, we revealed that SVCV regulated of the three UPR pathways during the infection (**Figure 10**). It is important to note that the viral modulation of the UPR is not universal, and some viruses can activate one specific pathway while others inhibit it at the same replication stage (34). TM treatment has been classically used to activate the UPR in cell cultures, and it is frequently included for comparative purposes. However, each cell type responds uniquely to the ER stress inducers (48). We confirmed that the TM induced a classical sequential activation of the three UPR pathways in the ZF4 cells. The ER stress and the activation of the UPR are frequently monitored by several methodologies such as western blotting and immunohistochemistry for UPR target genes, reporter assays for activity of XBP1 and ATF6, detection of IRE1α activation and ATF6 translocation to the nucleus with fluorescent microscopy (48, 49). In the present work, we determined the modulation of the different pathways by qPCR, measuring the expression levels of genes induced after the translocation of the ATF4, ATF6 and short XBP1 to the nucleus, as it has been validated in several publications (50).

During the early phase of infection, SVCV suppressed the expression of the BiP gene, as it has already been described in rhabdovirus like VSV and Maraba virus (8) and other viral groups such as the Porcine Reproductive and Respiratory Syndrome virus (PRRSV) (51) or Dengue virus (DENV) (52). This initial inhibition could retard the activation of the UPR until the viral genome was replicated and the amount of newly synthesized viral proteins was high. However, at the late phase of infection, the BiP gene was highly expressed, suggesting a critical role during the release of the progeny virions. BiP protein facilitates the assembly of viral components and its depletion results in an impaired budding or immature virion with diminished infectivity (53). BiP protein can be translocated to the cell membrane as a viral receptor or associated with mature virions to enhance their infectivity as an accessory host factor (16). Our results highlights that the precise control of BiP expression may allow SVCV to regulate when the UPR is activated (**Figure 10**).

This bi-modal modularity kinetics was also observed in the PERK pathway. Viruses such as DENV, hepatitis C virus, or human papillomavirus also followed this pattern (54). The low expression of the initial PERK and ATF4 genes and the downstream genes CHOP and GADD34 suggested a low modulation of this pathway at the early phase of infection that could allow the virus to override the host cell-mediated shutoff of protein synthesis and ensure a complete genome multiplication and viral protein assembly. Although we did not check the phosphorylation levels of the eIF2α, this pathway is activated by SVCV at the late phase of infection since the downstream genes GADD34 and CHOP were highly expressed. This is highly plausible since both genes are critical for the cellular response to viral infection (54). The increased expression of GADD34 contributes to the dephosphorylation of eIF2α and the recovery of the normal protein synthesis (55) and also induces the production of type‐I IFN and pro‐inflammatory cytokine during the late phase of an infection (56, 57). CHOP protein is also involved in essential processes during late viral infection. Although CHOP is mainly responsible for the life-or-death decision in infected cells with DNA and RNA viruses and mediates the activation of apoptosis and autophagy during microbial infection (58, 59), it also regulates the immune response in inflammatory processes through the regulation of cytokine expression (60). In this context, the high expression of the CHOP gene at late SVCV infection matches in time with the induction of autophagy and apoptosis at the end of the viral cycle, which is completely necessary during the release of nascent SVCV (61, 62) (**Figure 10**).

The ATF6 pathway is also targeted by several viral infections (34). Its activation benefits viral replication by increasing the chaperone expression to ensure the correct folding of viral proteins and to prevent protein aggregation (63). Moreover, ATF6 signaling can promote cell survival and inhibition of the innate immune response, as was described in the West Nile virus infection (64). However, the importance of this pathway for other viruses is still unclear. Surprisingly, SVCV does not modulate the ATF6 pathway under our infection conditions. Our data align with Isler et al. (63) and Gao et al. (51), who reported a suppression of the transcription factor ATF6 in cells infected with the Human cytomegalovirus and the PRRSV, respectively. Moreover, Jheng et al. (65), described that during enterovirus A71 infection, the transcription factor ATF6 was not translocated to the nucleus and its downstream target genes were not activated. It could be that SVCV increases the expression of alternative chaperone genes in an ATF6-independent mechanism as described for PRRSV infections (51). However, this hypothesis should be experimentally confirmed (**Figure 10**).

The most conserved UPR branch triggered by viral infections is the IRE1α pathway (34, 66). Activated IRE1-dependent signaling is detrimental to viral propagation through the degradation of viral RNAs and proteins by RIDD and ERAD, respectively (34). Moreover, Hinte et al. (67) described an unexpected role of the XBP1 as a potent repressor of both XBP1short and ATF6-mediated activation to inhibit viral gene expression and replication of human herpesvirus. However, the IRE1α pathway may also be beneficial to viral propagation. For example, this pathway favors flavivirus replication by increasing protein and lipid biogenesis and secretion of virions (66) or promoting the Hepatitis C virus by inhibiting apoptotic death of infected cells (68). Our data suggest that the IRE1α pathway could be activated at the late stage of infection when high levels of the total XBP1 gene and the downstream EDEM1 gene were detected. Since EDEM1 participates in the degradation of glycosylated proteins (69) and all enveloped viruses, including rhabdovirus, contain glycosylated envelope proteins (70), it is plausible that the ERAD pathway is activated in ZF4 cells at late stages of SVCV infection to modulate viral replication. However, the specific relationship between the ERAD pathway and SVCV viral replication should be analyzed and compared with that induced by other enveloped viruses (71) (**Figure 10**).

The inhibition of the UPR by SVCV was also confirmed by a second experimental approach previously described in other rhabdovirus (8). The UPR genes induced by TM were dramatically blunted when cells were previously infected with SVCV. We also evidenced that an active SVCV replication rather than a virus attachment inhibits the UPR. The UV-inactivated virus could not modify the UPR gene expression profile in both controls and TM-treated cells. This need for an active viral replication was also described in Zika virus infection using a UV-inactivated virus (72). Moreover, the low expression of the UPR genes in cells overexpressing the G and M viral proteins suggests that the generation of functional virions rather than the overexpression of a single viral protein is needed to interfere with the cellular UPR.

In summary, our data suggested that the active replication of SVCV inhibits the three arms of the UPR during the early phase of infection. However, the BiP gene and the downstream effectors included in the PERK and the IRE1 pathways were highly active at later stages. The ATF6 pathway and the downstream induced chaperone genes have minor participation during viral replication. Transcriptionally activated downstream genes could control other critical cellular processes such as apoptotic cell death, membrane biosynthesis, and host immune responses to favor viral replication (**Figure 10**).

Pharmacological remodeling of the UPR response is being explored as an alternative strategy to treat viral infections. However, special attention must be taken to avoid affecting endogenous protein maturation or causing toxicity (22, 37, 38, 66). It is necessary to analyze when and how the treatments are applied to get their maximum effectiveness. The antiviral activity of the chemicals was investigated using two complementary techniques, as proposed by Dolskiy et al. (73). Cell-based protocols complemented the PCR-based methods that detect viral genome replication. The multiple titrations of infected cellular supernatants allowed the detection of viable viral particles and were also used to correlate the viral concentration with the life cycle time.

The activation of the UPR by TM treatment inhibited SVCV infection only when cells were pretreated with the inducer or when cells were immediately infected after TM treatment. SVCV was not affected by the TM treatment once the infection had been established. Those observations suggest that the UPR can regulate SVCV infection only if activated during the early phase of infection and when the UPR effectors can limit viral replication. SVCV activates anti-UPR mechanisms at later infection stages to accommodate this cellular response into its life cycle. This time-dependent antiviral effect of the TM has also been described in other viral infection models, such as Zika virus (72) or West Nile virus (74).

However, as in other viral infections (66), we observed that the inhibition of the UPR can also negatively impact SVCV viral replication. The role of PERK during SVCV replication was assayed by using the GSK414. The treatment of ZF4 cells with GSK414 did not inhibit viral genome replication at 24 hpi suggesting that phospho-PERK alone does not have any effects on viral replication at least during the early stage of infection, in agreement with results from other viral groups and also using other PERK inhibitors (72). Although we have not checked the phosphorylation levels of PERK, the GSK414 was used in doses that inhibit entirely the PERK activity (75). We also specifically modulated this pathway by using GBZ. It significantly increased the ATF4 expression in ZF4 cells since it avoids the dephosphorylation of the eIF2α increasing the activity of the ATF4 and the downstream induced genes (66). However, when TM-induced UPR, GBZ treatment also affects the IRE1α pathway by increasing the expression of total and spliced forms of XBP1, highlighting that its mechanism of action is poorly understood. GBZ modulate the redox state by inhibiting the nitric oxide synthase (76) and alleviates the symptoms of human diseases such as neurodegenerative hereditary spastic paraplegia or amyotrophic lateral sclerosis by reducing the levels of ROS (77, 78). Moreover, GBZ can reduce pro-inflammatory responses (79). GBZ significantly affect the replication of the SVCV genome during early infection stages and delays the assembly and release of the new viral progeny at later stages, as the late appearance of CPE suggests. Possible antiviral effects of the GBZ could be due, at least in part, to its ability to maintain physiological processes involving free radicals that are well balanced within the host. A similar antiviral mechanism has been proposed for several natural products where enhanced antioxidant enzyme activities and decreased reactive oxygen species (ROS) inhibit SVCV infection (80). The use of GBZ as an antiviral agent should be further analyzed, moreover when it has antiparasitic activity against toxoplasmosis (81).

To evaluate the role of the IRE1α pathway in SVCV replication, we employed two complementary compounds that target the same pathway: 4μ8C and APY29. The treatment with the kinase inhibitor APY29 induced significant changes in the viral cycle of SVCV, while the endonuclease inhibitor 4μ8C did not affect it. The reduction of genome multiplication during the early stage of infection was accompanied by a delay in the assembly and release of new viral particles, as suggested by the late appearance of CPE in treated cells. Similar antiviral activity of the APY29 has already been described in HEC-1-A cells infected with Herpes simplex virus type 1 (HSV-1) (82) suggesting that the kinase activity of IRE1α favors SVCV replication. Although APY29 has been described as a specific allosteric modulator of IRE1α by inhibiting the IRE1 autophosphorylation (27), our qPCR data suggest that this inhibitor affects the three pathways of the UPR. As expected, the blockage of the IRE1α phosphorylation reduced the spliced XBP1 gene, affecting the expression of EDEM1 and DNAjC3. However, the ATF6 and PERK-ATF4 pathways were also inhibited.

The modulation of the ATF6 pathway also affected the SVCV replication. We confirmed in our ZF4 cell model that ceapin A7 has low cell toxicity and specifically blocks the ATF6 pathway, which was previously described in other publications (26). Our results highlight that ceapin A7 only exerts its inhibitory and antiviral activity when present in the medium; moreover, this compound can be washed out from the cell by removing it from the cell culture medium. Ceapin A7 not only significantly decreased the replication of the viral genome but also delayed the appearance of CPE and viral load during the late infection stages. This ability of ceapin A7 to inhibit viral replication at late infection stages has already been described in the Zika virus (72). It suggests that the ATF6 pathway is required for the late phase of infection.

Next, we confirmed that the combination of the modulators would have a cumulative effect on SVCV replication. As we described before, the highest antiviral activity induced by APY29 resulted from the unspecific modulation of several UPR pathways. The joint modulation of PERK and ATF6 pathways significantly decreases the SVCV replication. Similar combined attenuation of the PERK and ATF6 pathways has also been described to inhibit ZIKV replication (72). Combined treatments with additive or synergistic effects on the UPR have also been explored in suppressing SARS-CoV-2 replication (83). However, it is needed to evaluate whether this combination therapy increases cytotoxicity. Further experimental studies are required to understand the relative importance of each UPR arm in SVCV infection as an essential step in developing a combinatory antiviral treatment.

A preliminary screening of each modulator’s *in vivo* antiviral activity was conducted in zebrafish larvae. Only TM, GBZ and ceapin A7 were used since they have already been administered in zebrafish to analyze activities not related to viral infections such as protective effects against neurodegenerative diseases (hereditary spastic paraplegia and amyotrophic lateral sclerosis) or induction of steatosis in the liver (77, 84–86). The other compounds, like 4μ8C and APY29, were not used since they are unsuitable for systemic administration. Their pharmacokinetic properties and side effects limit their usefulness in animal studies (26, 28, 29). The toxicity of the treatments was evaluated by the lack of morphological changes and the induction of mortalities. Conducting specific pharmacodynamics studies in fish is important since non-lethal histopathological lesions can be induced without showing any external clinical signs. This is the case of the TM treatment that shows a potent liver toxicity in fish at nontoxic concentrations (87). Although TM is a promising drug in chemo-and immunotherapy, its direct administration in mice models is not adequate since TM’s residual cytotoxicity affects surrounding tissues around a tumor (88). The side effects of GBZ have already been extensively analyzed in humans since this drug was approved for the treatment of hypertension (25). In humans GBZ decreases the heart rate and relaxes the blood vessels so that blood can flow more easily through the body. Moreover, it can affect the excretory activity of the kidney by enhancing the water diuresis (89). Despite kidney in both fish and mammals shares some excretory functions, they show significant differences in structure and other physiological functions, reflecting adaptations to their respective aquatic and terrestrial environment. However, specific studies must be conducted to analyze the effects of modulators on fish physiology.

The *in vivo* antiviral activity of those compounds has never been tested before. Under our experimental conditions, treating fish larvae with TM, GBZ and ceapin A7 did not confer any protection against a lethal SVCV infection. Moreover, the mortality kinetics were faster in fish previously treated with the modulators, suggesting that uncontrolled side effects could be induced in fish, increasing the susceptibility to the viral infection. However, the antiviral activity of the GBZ and ceapin A7 cannot be excluded. It must be analyzed in detail (fish age, dosage, route of delivery, duration of the treatment) since additional properties such as anti-inflammatory, anti-obesity and calming activity have been described in other animal models (90–92).

In conclusion, our results indicate that SVCV modulates the three branches of the UPR signaling pathways during infection in a time-dependent manner, and SVCV reprograms the UPR of the host to its advantage. Using modulators is an effective strategy for inhibiting SVCV infection *in vitro*. We also provide a better understanding of virus-host interactions and suggest an attractive and practical approach for designing antiviral therapeutics against SVCV. However, the *in vivo* anti-viral activity of UPR modulators against SVCV infection needs to be further validated. We speculate that this anti-viral activity results from the combination of several molecular pathways interlinked with the UPR that results in the inhibition of viral replication. However, this combined effect needs to be analyzed in future studies.

## Data Availability

The raw data supporting the conclusions of this article will be made available by the authors, without undue reservation.
