# Supplementary material for: Spring viraemia of carp virus modulates the time-dependent unfolded protein response to facilitate viral replication
Source: Front Immunol. 2025 Apr 3;16:1576758. doi: 10.3389/fimmu.2025.1576758 (PMC12003378; doi:10.3389/fimmu.2025.1576758)

Supplementary Material

**Supplementary Figure 1:** The toxicity of the different UPR modulators was evaluated by analyzing the morphological changes by light microscopy and the cellular metabolic activity by the MTT assay. The cells were treated with the different modulators for 24 h and then removed from the medium. The toxicity was evaluated at 24 h, 48 h, 72 h and 6 days after treatment. An example of the morphological changes observed by light microscopy in cells treated with TM after 6 days post stimulation is included. Scale bar = 100 μm. Representative results obtained in the MTT assay 24 h after treatment with TM and ceapin A7 are also presented. The significant reduction of the O.D. at 560 nm indicates a toxic effect on the cells. Data were analyzed by one-way ANOVA. Significant differences between TM treated and control cells were analyzed by a Tukey’s post hoc test and represented by * (*P*<0.05).


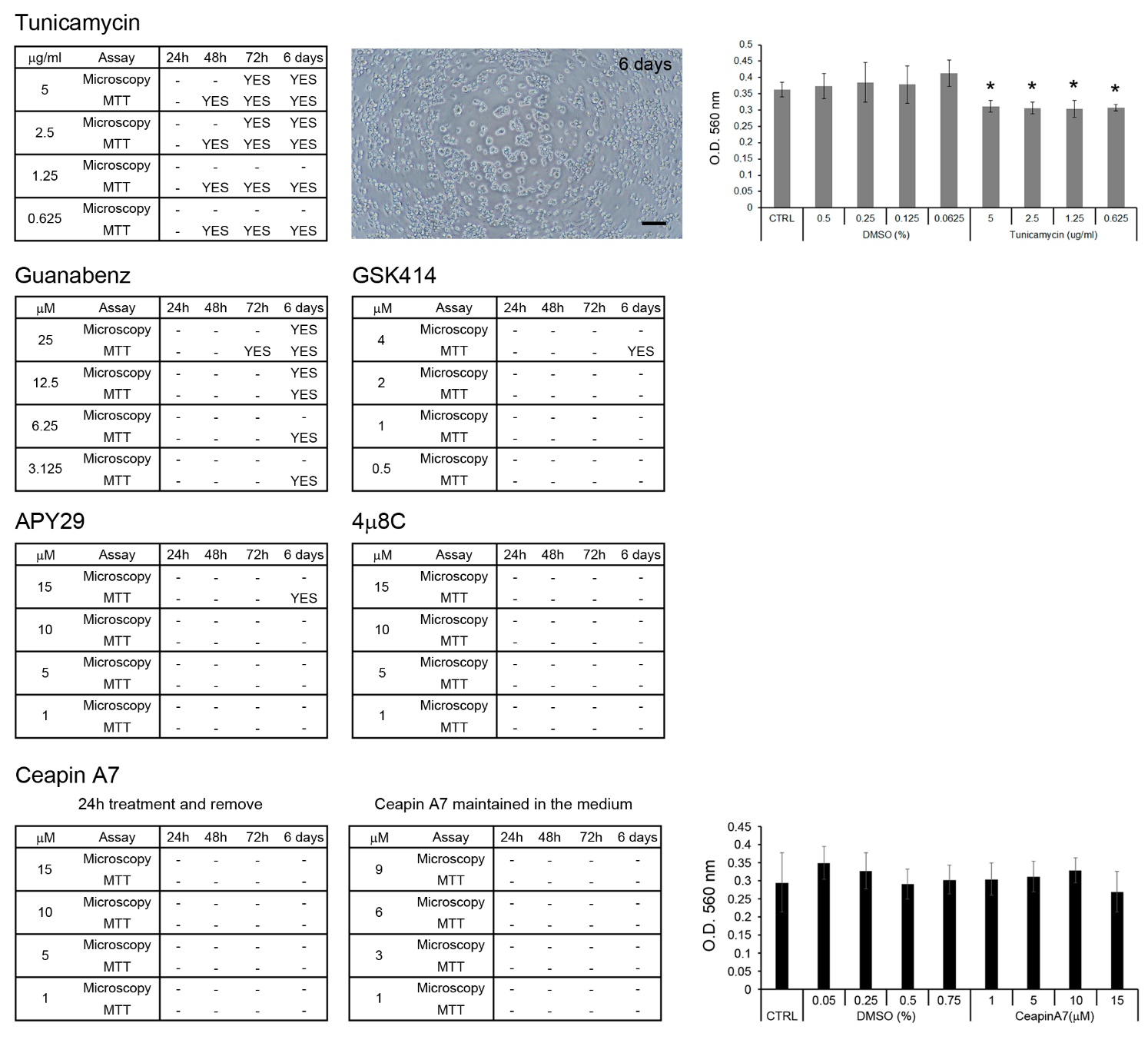


**Supplementary Figure 2:** Specificity of the GBZ (12.5 µM) to modulate the PERK pathway. The expression profile of all UPR genes was evaluated by qPCR. Results represent the mean and SD of four independent samples. Data were analyzed by one-way ANOVA. Significant differences between GBZ treated and control cells were analyzed by a Tukey’s post hoc test and represented by * (*P*<0.05) and ** (*P*<0.01).


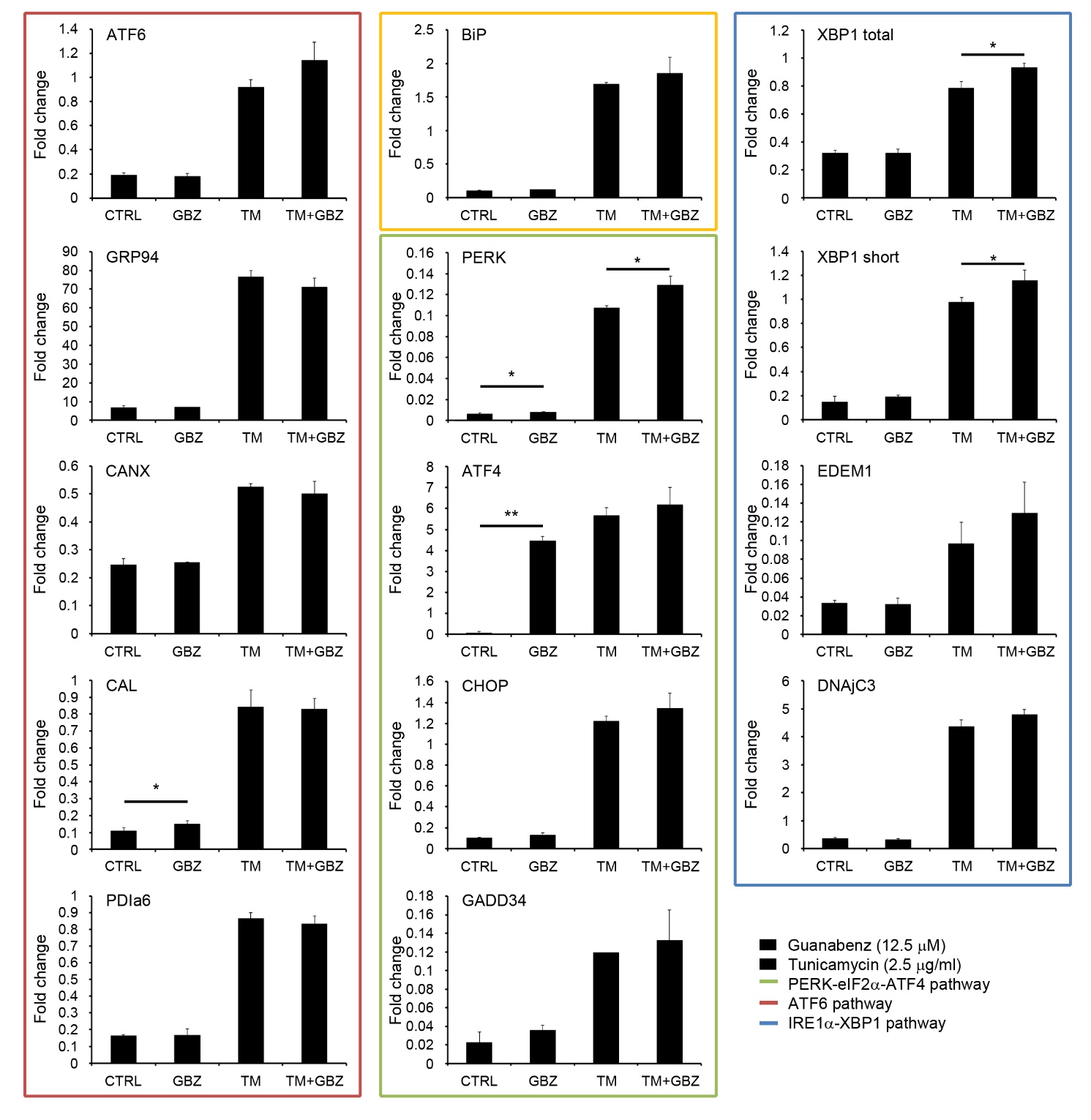


**Supplementary Figure 3:** Specificity of the APY29 (2 µM) to modulate the IRE1α pathway. The expression profile of all UPR genes was evaluated by qPCR. Results represent the mean and SD of four independent samples. Data were analyzed by one-way ANOVA. Significant differences between APY29 treated and control cells were analyzed by a Tukey’s post hoc test and represented by * (*P*<0.05) and ** (*P*<0.01).


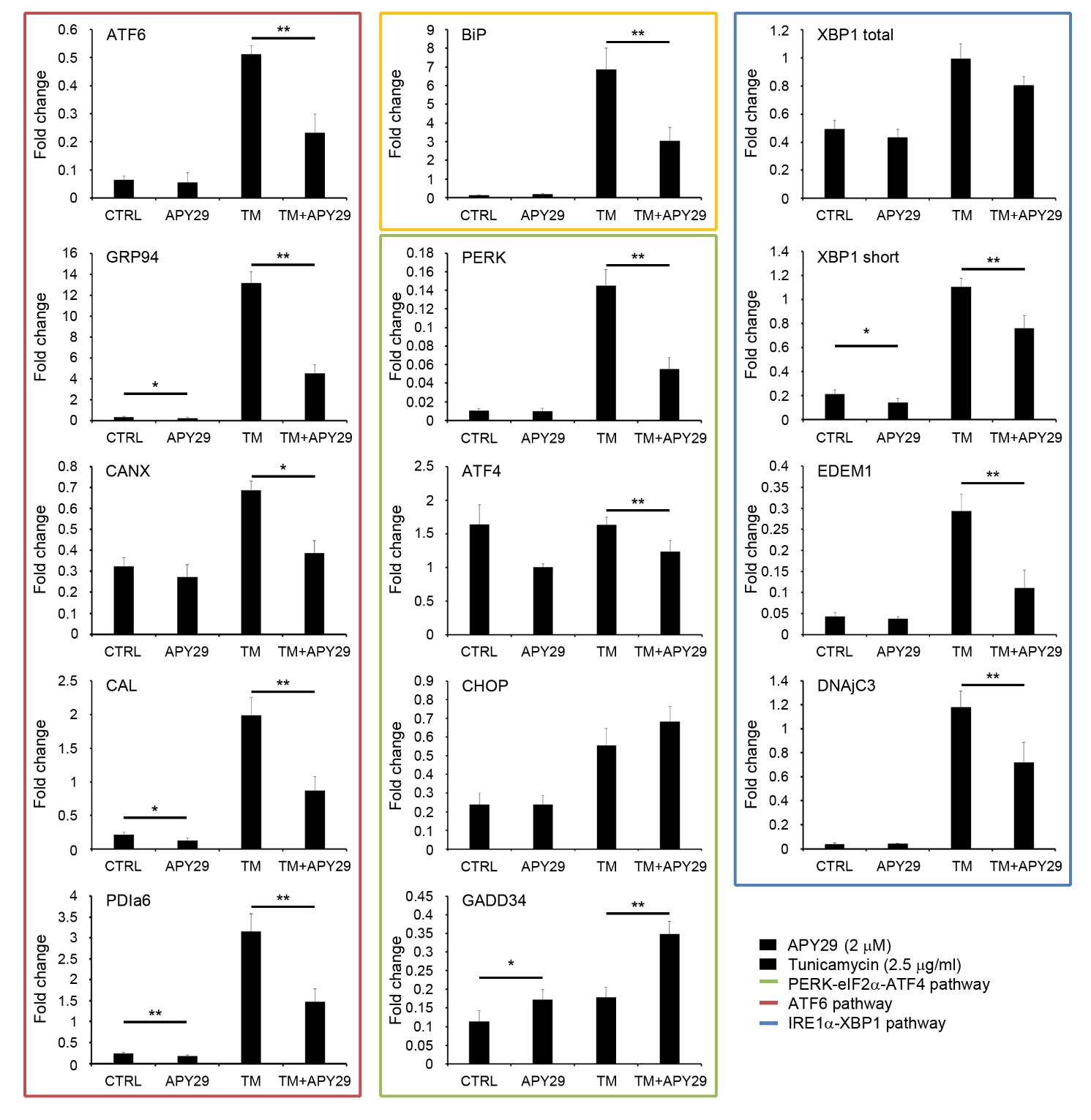


**Supplementary Figure 4:** The specificity of the ceapin A7 (15 µM) to modulate the ATF6 pathway was evaluated by qPCR. Results represent the mean and SD of four independent samples. Data were analyzed by one-way ANOVA. Significant differences between ceapin treated and control cells were analyzed by a Tukey’s post hoc test and represented by * (*P*<0.05) and ** (*P*<0.01).


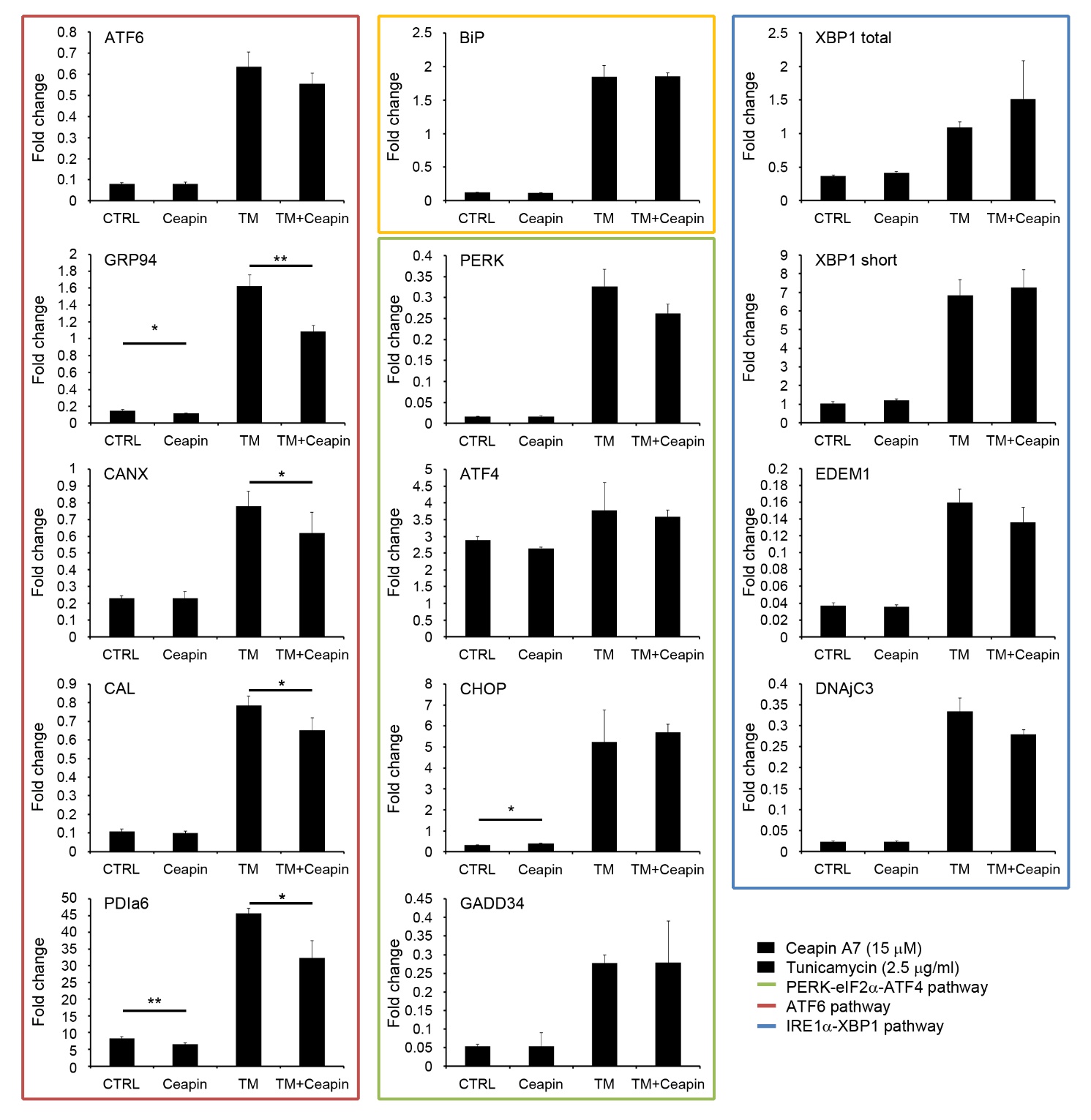

Supplement: Supplementary file 1 [file DataSheet1.docx]
